# Nanostructured plasmonic metapixels

**DOI:** 10.1038/s41598-017-08145-0

**Published:** 2017-08-10

**Authors:** Calum Williams, Girish Rughoobur, Andrew J. Flewitt, Timothy D. Wilkinson

**Affiliations:** 1 Centre of Molecular Materials for Photonics and Electronics, Electrical Engineering Division, Department of Engineering, University of Cambridge, 9 JJ Thomson Avenue, Cambridge, CB3 0FA United Kingdom; 2 Electronic Devices and Materials Group, Electrical Engineering Division, Department of Engineering, University of Cambridge, 9 JJ Thomson Avenue, Cambridge, CB3 0FA United Kingdom

## Abstract

State-of-the-art pixels for high-resolution microdisplays utilize reflective surfaces on top of electrical backplanes. Each pixel is a single fixed color and will usually only modulate the amplitude of light. With the rise of nanophotonics, a pixel’s relatively large surface area (~10 *μ*m^2^), is in effect underutilized. Considering the unique optical phenomena associated with plasmonic nanostructures, the scope for use in reflective pixel technology for increased functionality is vast. Yet in general, low reflectance due to plasmonic losses, and sub-optimal design schemes, have limited the real-world application. Here we demonstrate the *plasmonic metapixel*; which permits high reflection capability whilst providing vivid, polarization switchable, wide color gamut filtering. Ultra-thin nanostructured metal-insulator-metal geometries result in the excitation of hybridized absorption modes across the visible spectrum. These modes include surface plasmons and quasi-guided modes, and by tailoring the absorption modes to exist either side of target wavelengths, we achieve pixels with polarization dependent multicolor reflection on mirror-like surfaces. Because the target wavelength is not part of a plasmonic process, subtractive color filtering and mirror-like reflection occurs. We demonstrate wide color-range pixels, RGB pixel designs, and in-plane Gaussian profile pixels that have the potential to enable new functionality beyond that of a conventional ‘square’ pixel.

## Introduction

High resolution reflective microdisplays, based on liquid-crystal-on-silicon (LCoS) technology, are ubiquitous, underpinning a plethora of optical modulation applications including projectors, telecommunication switches, head-mounted displays^1–3^ to name but a few. A single pixel typically consists of linear polarizers, RGB pigment-based color filters and an electrically switchable waveplate (liquid crystal layer) sitting on top of a mirror-quality reflector (a square aluminum electrode ~10 *μ*m^2^) connected to electronic circuitry^1, 3^. The purpose of this surface is purely to reflect the incoming light. However, with the simultaneous rise of nanofabrication capabilities and utilization of the unique optical phenomena associated with plasmonic devices^4–6^, there exists an opportunity to exploit this relatively large metallic surface area in order to significantly increase the functionality of microdisplay pixels with nanophotonics. Pixels capable of modulating not only amplitude, but polarization, wavelength and even the ability to adjust their in-plane shape, have potential applications ranging from 3D holographic displays, that fully represent the plenoptic optical wavefront^7, 8^, to hyperspectral structured illumination microscopy^9–11^, requiring sample illumination across different wavelengths and polarizations. Surface plasmons (SPs) can be considered light waves trapped on a metal surface^4^ and can be used to localize light to dimensions far below the optical wavelength^12–14^. Subsequently, plasmonic nanostructures have attracted a wealth of interest over the past few decades leading to a myriad of potential optical applications including sub-wavelength spectral filters^15, 16^, nanoscale plasmonic waveguides^17^ and holographic display elements^18–21^. For reflective pixel purposes, the attraction of plasmonics lies in sub-pixel control of the light including polarization enhanced spectral filtering and the patterning of complex information directly on a pixel’s surface. Initially, this may alleviate the need for pigment filters and incident polarizers, yet *engineered pixels* offer the potential to re-imagine the way in which one considers what is meant by a single pixel. Specifically, a pixel now has the ability to encode wavelength, polarization, phase and amplitude information directly on its surface^7, 8, 20, 22, 23^. This marks a step towards fully representing the plenoptic function of the light field, a requirement for future display devices^7^. Recent studies have highlighted the shortcomings of current plasmonic pixel technologies, and include: sub-optimal design schemes resulting in a low optical amplitude^23–27^, due to inherent loss mechanisms within color-generating metallic nanostructures; small color gamuts due to the type of plasmonic resonance utilized (i.e. poor full-RGB coverage)^16, 24, 25, 28, 29^; structures using materials incompatible with industrial manufacturing techniques and long-term use in real-world devices, such as photoresist^28, 30^; and focusing on the patterning of a fixed image on the surface (lack of reconfigurability)^28, 30^, which is completely ineffectual for any real world display system that must dynamically update. We overcome these shortcomings through multimodal absorption in nanostructured plasmonic metal-insulator-metal (MIM) pixel designs: *metapixels*. Our solution offers near-perfect reflection for the target color, wide wavelength selectively, polarization switchable sub-pixel optical properties and compatibility with industrial manufacturing techniques.

Generally, MIM structures are tailored for applications requiring near-perfect absorption of a particular wavelength range with near-perfect reflection of other wavelengths^31, 32^. Previously, the strong absorption mode associated with the structures presents a problem for wide-color gamut/RGB reflective pixels^28, 30^. In this work, by combining plasmonic nanostructures with MIM geometries, we can excite multiple modes simultaneously in order to tailor the optical properties of a reflector. We demonstrate new reflective pixel designs based on plasmonic nanostructure MIM geometries which offer highly reflective, polarization dependent, color filtering in the visible spectrum (400–700 nm). The devices suppress the unwanted colors either side of the target wavelength through the combination of the absorptive SP and plasmonic waveguiding modes (guided mode resonance). Hence, due to MIM geometry, mirror-like reflection for the target wavelength, and strong absorption for the unwanted wavelengths occurs. In addition, 1D, 2D and nanostructure arrays allow for the coupling of only certain polarization states and wavelengths to the absorption modes. We extend this concept to create more advanced pixels which incorporate in-plane 2D amplitude functions on the pixel itself, encoded with nanostrucutres. We use Gaussian-profile plasmonic pixels for the spatial tailoring of the color properties on a sub-pixel basis. A design whereby pixels are now no longer just rectangular, and moreover, each pixel has polarization controlled color spatial functions, ideal for the integration with liquid crystals (switchable waveplates). This represents a departure from the conventional paradigm of using square pixels that only encode amplitude.

To achieve specific color properties, we use Ag(30 nm)-SiO_2_(100 nm)-Al(100 nm)-Si(bulk) layers. Aluminum is typically the material of choice for back-reflectors in microdisplays with silver an excellent and widely used plasmonic material across the visible. SiO_2_ is cheap, mechanically and chemically stable, and has relatively flat dispersion across the visible. Hence the devices presented in this work are an exciting prospect for integration with LCoS pixel technology, which includes multiple polarizers and incorporates pigment-based color filters for spectral filtering. By varying periodicity, grating widths and designs, a range of polarization dependent plasmonic MIM color pixels are produced which utilize the existing Al reflectors as the back-reflector in a typical MIM-pixel backplane. The work here can eliminate the need for additional polarizers and pigment-based color filters on the display backplane, and adds additional functionality through polarization-spectral control and in-plane pixel color functions. Moreover, the dimensions and materials utilized means the designs are highly compatible with a range of methods for larger scale manufacturing, including extreme-UV photolithography and nanoimprint lithography^33–35^.

## Results

**Figure 1:**
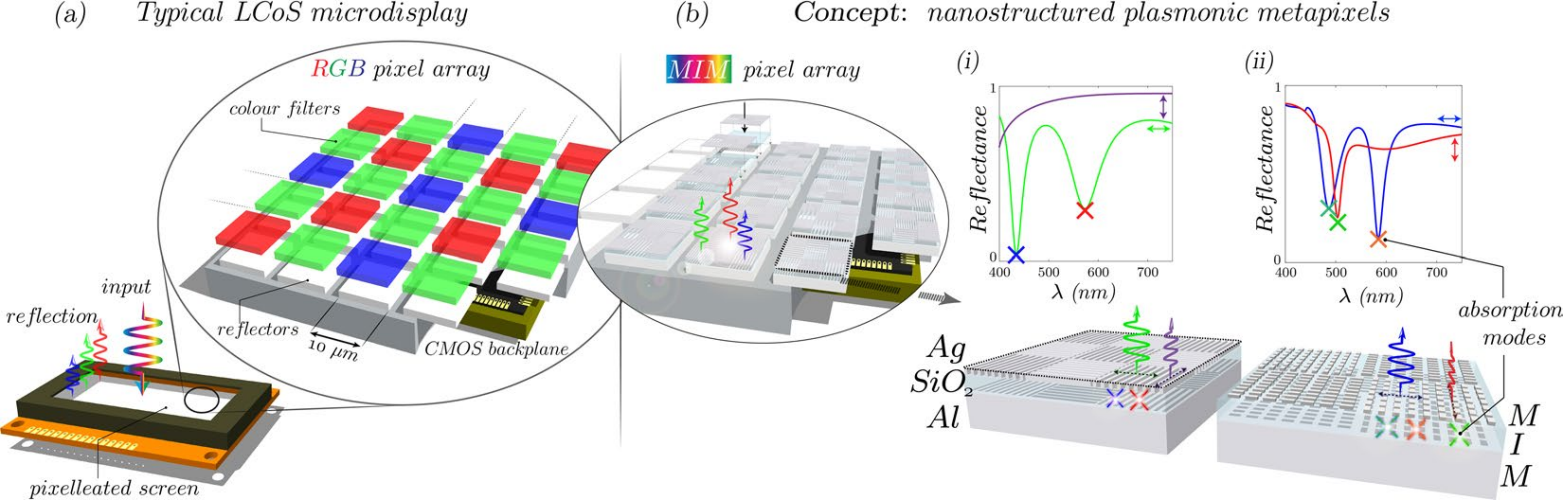
Plasmonic MIM pixel (metapixel) concept. (**a**) Reflective LCoS microdisplay: schematic example of typical RGB-filter pixel array reflectors. Relatively large, pigment-based filters control color and hence each pixel can only be one color. From the conventional pixel to (**b**) the nanostructured plasmonic metapixel concept; demonstrating modulation of color. The designs are based on nanostructuring the available reflector area whereby for each target wavelength, a minimum of two absorption modes are tailored to sit either side of this wavelength thus eliminating the requirement for RGB filters in (**a**). The proposed pixel designs (i) and (ii), represent both 1D (sub-pixel 1D gratings) and 2D nanostructuring (sub-pixel isolated nanostructures) respectively, for achieving the different polarization dependent optical properties shown in the simulated reflection spectra above the schematics. For the 1D case (i), color modulation or near-perfect reflection can be controlled. For the 2D case (ii), multi-color modulation can be achieved. Hence now, pixels can include multiple state and multiple color functionality, yet still offer high reflection (due to MIM geometry).

In comparison to a conventional LCoS microdisplay Fig. 1(a), our proposed plasmonic metapixels, schematically shown in Fig. 1(b), utilize several key resonant phenomena to form their unique optical response. Through nanostructured periodicity (both 1D (b-i) and 2D (b-ii)), providing an additional in-plane momentum component, SPs can be excited at normal incidence: both propagating (PSPs) or localised (LSPs), depending on the geometry^12^. Anisotropic geometry enables polarization dependency, and shrinking features to sub-wavelength, leads to color filtering. Integration into MIM geometry results in the plasmonic quasi-guided modes (QGMs) and cavity modes (CMs) being excited^13, 36^. For the former, the structure acts as a plasmonic waveguide for wave propagation tangential to the interface^17, 31, 37, 38^. Hence, metapixels, unlike conventional pixels, have optical functionality (color, polarization, amplitude, in-plane functions) encoded with nanostructures (or sub-pixels) on the pixel reflector itself. To excite a waveguide mode, top-layer periodicity (nanostructuring) is utilized - analogous to an out-of-plane grating coupler. The nanostructured top-layer grating scatters light into multiple modes, with varying wave-vector components. It is then possible to couple these diffracted modes into waveguide modes, albeit leaky, which propagate tangential to normal incidence. Moreover, depending on insulator thickness, plasmonic and or oscillatory (conventional) waveguiding modes can be excited^13, 36, 39^. The former (exploited in this work) has tangential E-fields confined to the interface. In addition, as the number of possible excitation modes increase, resonant-modes overlap, are simultaneously excited, and hybridization occurs. The modes in the devices in this work consist of hybridized forms of SP modes, CMs and QGMs (plasmonic).

### MIM grating pixels

**Figure 2:**
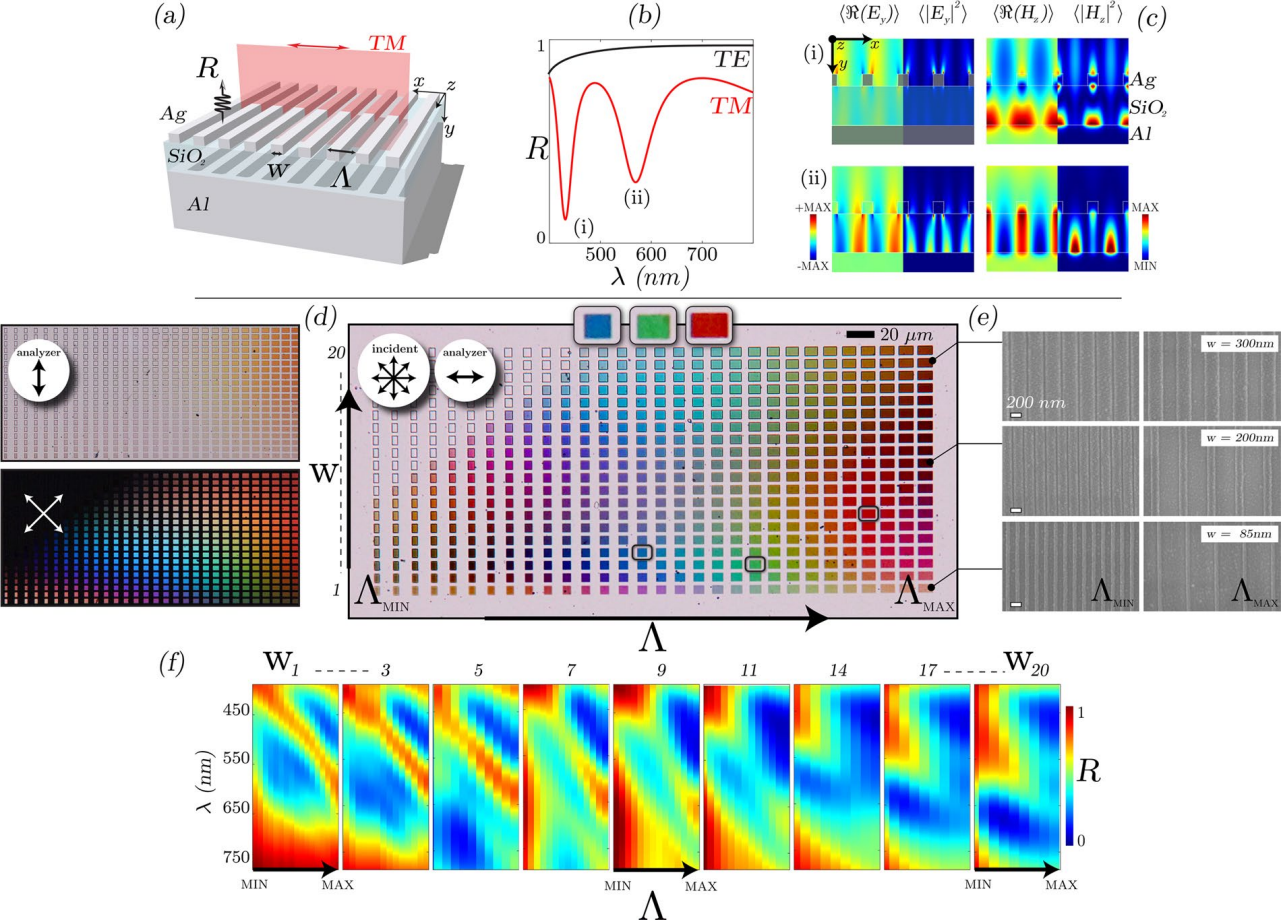
1D grating plasmonic-MIM pixel: (**a**) Schematic of 1D MIM grating with common grating parameters defined: grating widths, *w*_*g*_ (nm), grating period, Λ (nm), duty cycle, Γ = *w*_*g*_/Λ. (**b**) Shows the simulated reflection response of a typical 1D plasmonic MIM pixel with two resonant absorption modes, with the associated field profiles shown in (**c**). A more detailed set of simulations is found in the Supplementary Material. (**d**) Optical characterization results of a range of pixels exhibiting vivid colors under varying polarization conditions and (**e**) the SEM images of the min and max gratings for several rows. In (**d**), the three insets are zoomed in versions of selected RGB colors in the larger array. (**f**) Experimental reflection measurements, *λ* vs. Λ (which varies in the x-axis in (**d**)), of different pixel rows (1–20), showing multiple distinct resonant mode (absorption) profiles, which increase in wavelength with increasing grating period. Between the two modes there is the region of high reflection, due to Al back reflector, which is spectrally dependent on grating width and grating period.

By combining ultra-thin (30 nm) plasmonic 1D gratings with MIM geometry, we now investigate our first pixel concept. Figure 2 shows the concept (a) and results of a matrix of 1D plasmonic MIM grating arrays with varying grating widths, *w*_*g*_ (nm), and grating period, Λ (nm), with duty cycle, Γ = *w*_*g*_/Λ. The pixels are patterned using electron-beam lithography (EBL), the back-reflector is made from sputtered Al (100 nm), the insulator is reactively sputtered SiO_2_ (100 nm) and the nanostructured top-layer is thermally evaporated Ag (30 nm) - full fabrication details described in the *Methods* section. These parameters are chosen to optimize for dual-resonant (SPP and QGM) operation within visible wavelengths. Specifically, the insulator thickness is set such that only the fundamental plasmonic waveguiding mode (QGM) can exist^13, 17, 36^, and Ag top-layer for optimal SPP excitation behavior. Extensive details and supporting simulations are shown in the Supplementary Material.

Crucially, each 1D reflector pixel, Fig. 2(a), exhibits a minimum of two resonances: the first, is a SP-mode and the second is a QGM. Figure 2(b) shows the typical reflection response and field profiles (c) of a nanostructured MIM-grating (*w*_*g*_ = 120 nm, Λ = 400 nm, duty cycle = 0.3) for TM-polarization from full-wave FDTD simulations^40^. The two absorption modes are highlighted: the shorter wavelength mode (~427 nm) - which is close to the fundamental vertical CM - exhibits SPP, CM and QGM field-profile characteristics^17, 31, 36, 37, 41, 42^; the longer wavelength mode (~575 nm) exhibits SPP (Ag-SiO_2_) and QGM characteristics. We provide a more in-depth explanation of layer thicknesses and origins of the modes in the Supplementary Material - with specific consideration to dispersion relations. The modal dispersion is such that the grating width and duty cycle variation leads to stronger or weaker coupling into the associated system modes.

**Figure 3:**
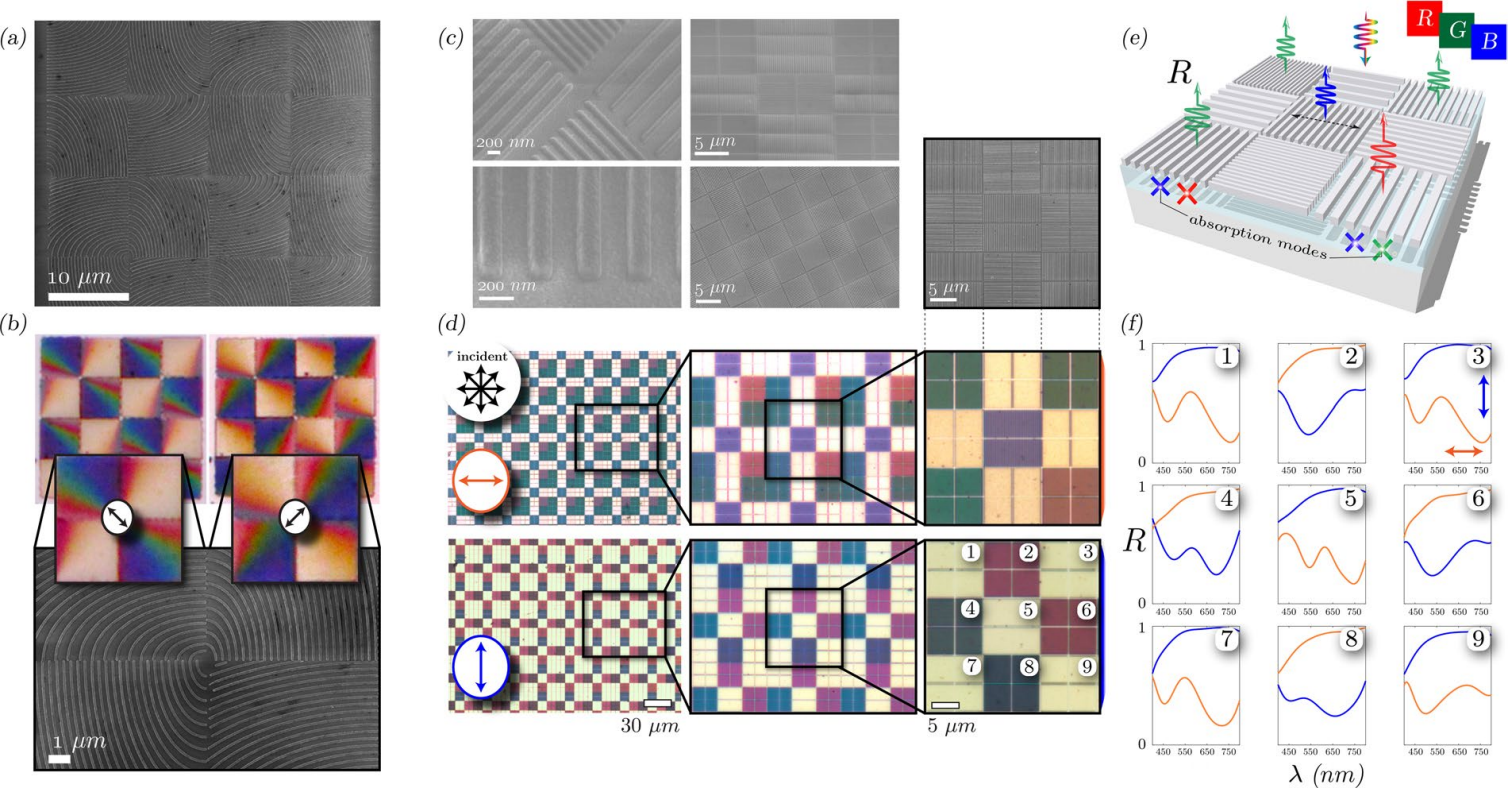
RGB plasmonic pixel design: (**a**,**b**) Gradient spacing spirals under SEM and optical microscope under two orthogonal polarizations conditions for the analyzer (unpolarized incident). (**c**) SEM images of RGB pixel design, schematically shown in (**e**), at varying magnifications. (**d**) Optical characterization of an array of plasmonic pixels exhibiting RGB colors under varying polarization conditions (normalized to Al mirror) and (**f**) the associated reflection measurements of the main unit cell with orthogonal polarization conditions. This displays either thin-film silver reflection or dual-mode resonant absorption (color filtering) depending on analyzer condition.

**Figure 4:**
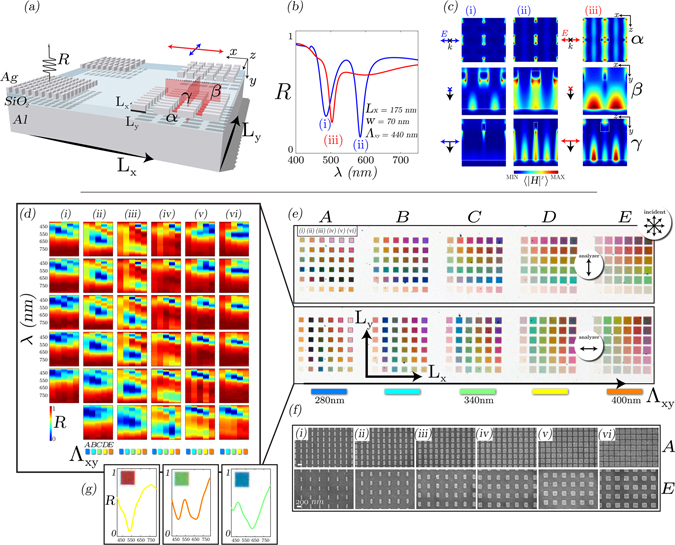
2D plasmonic-MIM pixel: (**a**) Schematic of nanostructured MIM plasmonic structures, with the nanostructure dimension in the x-dimension, L_*x*_, and y-dimension L_*y*_, with symmetric grating period, Λ_*xy*_. (**b**,**c**) Selected FDTD simulations (reflection response and associated field profiles) of a typical 2D plasmonic MIM pixel with two resonant absorption modes - taken from larger set of simulations shown in Supplementary Material. *α* is the x-z plane intersection, *β* the x-y plane and *γ* the z-y plane. (**d**,**e**) Experimental optical characterization results of a range of pixels exhibiting vivid colors under varying polarization conditions, with selected pixels exhibiting RGB behavior shown in (**g**). (**f**) SEM images showing the extremes of the range of different arrays; from smallest-to-largest grating period (A–E) and smallest-to-largest L_*x*_ parameter.

**Figure 5:**
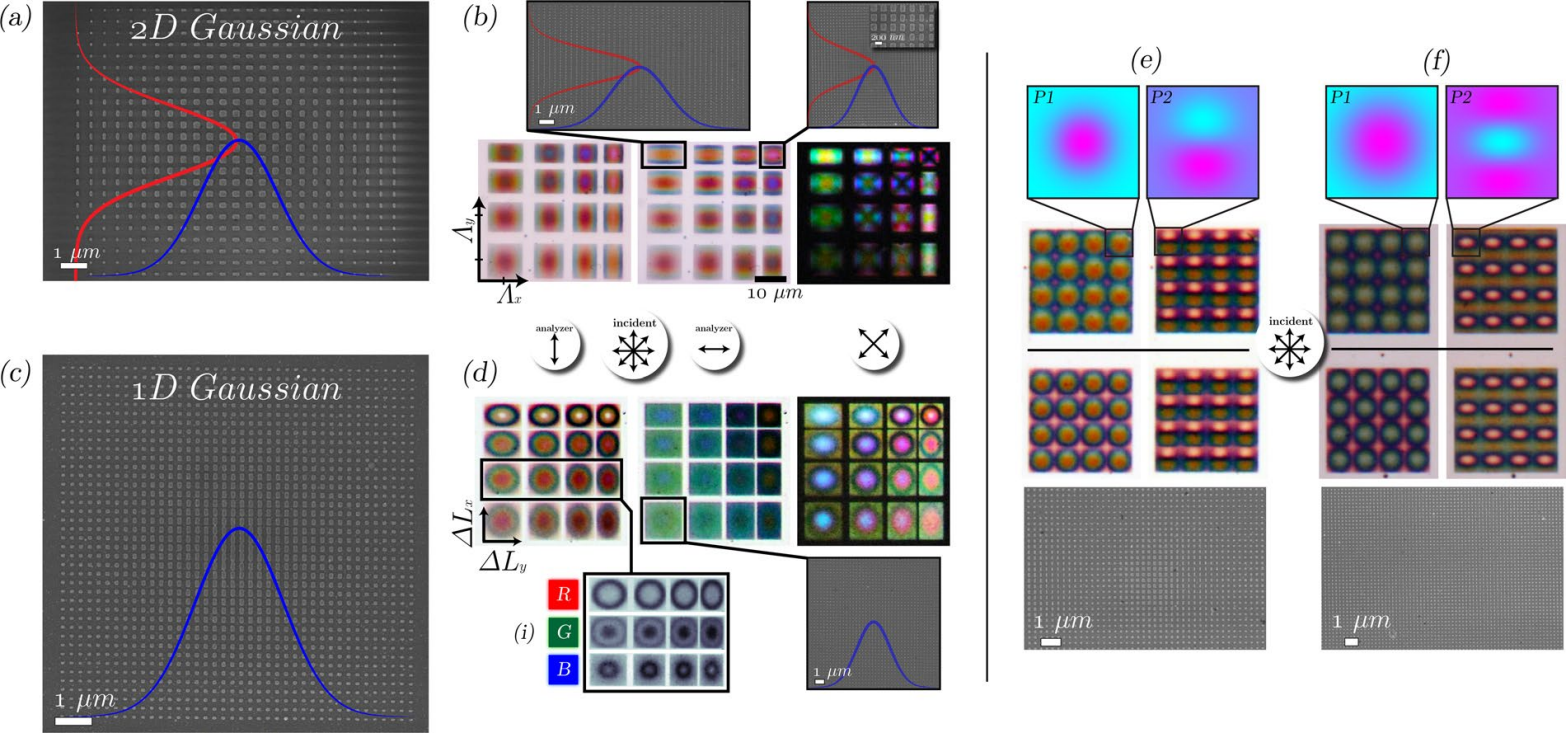
Gaussian plasmonic MIM pixels: (**a**,**b**) SEM and optical images (microscope) of 2D Gaussian nanostructured pixels, with the overlaid Gaussian functions in x/y. The optical images are with two orthogonal analyzer conditions and a crossed-polarization state, showing gradient color functions associated with each pixel. Λ_*x*_ and Λ_*y*_ is the grating period in x and y respectively. Δ*L*_*x*_ and Δ*L*_*y*_ are increase in the geometry of the nanostructures dictated by the Gaussian functions, where the total length of each structure is *L*_0_ + Δ*L*_*x*_ and *L*_0_ + Δ*L*_*y*_ for x-and-y dimensions respectively and *L*_0_ is the initial length, 60 nm (same for both axes). (**c**,**d**) As previous, but with a 1D Gaussian profile. The inset in (**d**), (i) is of the RGB channels of one of the Gaussian rows exhibiting how each pixel encodes varying responses depending on the wavelength. (**e**,**f**) Show the encoding of a 2D Gaussian with first-order partial derivative and second-order derivatives (Laplacian operator) for orthogonal polarization states.

Each grating is 10 *μ*m in length but with variation in total width. The designed grating width varies from ~80–310 nm and grating unit cell varies from ~180–600 nm. SEM images of the smallest and largest spacing pixels are shown in Fig. 2(e). For polarized incident light and analyzer aligned with TM-polarization (parallel to grating vector), a range of vivid colors in reflection are observed (d). The surrounding white part of this image is 100% reflection from the Al-back reflector. By varying the analyzer rotation the arrays transition from conventional reflector pixels to plasmonic-enhanced pixels, shown in (d). At a large grating period (low duty cycle), resonant spectra are observed. Figure 2(f) presents the experimental reflection spectra (*λ* vs. Λ) of a selection of the rows (labeled 1–20) as a function of duty cycle, in good agreement with simulations results (Supplementary Material). For all spectra, at least two modes are observed, which decrease in resonant wavelength as duty cycle increases (spacing decreases). In the spectra with narrowest grating widths, i.e. up to ~85–175 nm ((f) - sub-figures 1–7) strong, sharp, reflection peaks are obtainable in the visible part of the spectrum (~400–650 nm) with absorption modes either side. The reflection is normalized to a reference mirror, and due to MIM-geometry, efficiencies are high.

By selecting the widths and gratings which correspond to RGB color profiles, we can create sub-pixel arrays which act as polarization selective color pixel reflectors - shown in Fig. 3 (concept - e). Initially, we use gradually increasing spacing spiral design pixels (a-b), to investigate the color properties under varying analyzer conditions. These show bright color gradients from blue-to-red, which can be varied/switched with analyzer rotation. From this, the main RGB plasmonic-MIM pixel array concept is created - Fig. 3(c–f), which is loosely based on a typical Bayer color filter mosaic. Under incoherent, unpolarized illumination, orthogonal analyzer polarizations result in either broadband reflection or plasmonic color selection - shown in (d-f). For the latter, each sub-pixel exhibits absorption modes either side of their respective color - shown in the reflectance spectra in (f). With TE-mode selected (analyzer parallel to grating-vector), the reflectance tends to that of thin-film silver. Note that for plasmonic color selection, due to high reflectance of the target wavelength from the back-reflector, the reflectance tends to that of perfect linearly polarized light reflection i.e. 50%. The demonstration of the importance of MIM-geometry is shown in the Supplementary Material - experimental section.

### 2D plasmonic MIM pixels

2D nanostructured-MIM-pixel-arrays allow for additional degrees-of-freedom for polarization and spectral control, instead of just color or mirror-like reflection, as shown in the previous section. Moreover, in comparison to existing display technology, a pixel now has the ability to become more than just one color. Figure 4 shows the concept (a), and optical results (d–e) of the 2D plasmonic MIM reflector array. The total area for each sub-array is comparable to high-resolution reflective microdisplay pixels, yet here, color is generated without pigment, and amplitude response without the required incident polarization state. Selected simulation results are shown in (b–c) - highlighted from a comprehensive study described in the Supplementary Material. In general, the anisotropic nature of a typical MIM nanostructure leads to either dual-resonance behavior (orthogonal E-field to the long-axis) or single resonance (parallel E-field to the long-axis). We can identify both SPP and QGM behavior modes, which are dependent on unit cell periodicity in 2D and nanostructure geometry. The field intensity profiles (at the resonance positions) at various cross-sections across the array, are shown Fig. 4(b,c), are indicative of these modes^17, 31, 36, 37, 41, 42^.

Figure 4(d,e) shows the experimental results of the plasmonic MIM pixel arrays, with SEM images of selected pixels shown below in (f). The nanostructure arrays, which have dimensions ranging from ~75–250 nm in both the x-dimension (L_*x*_) and y-dimension (L_*y*_), with symmetric grating period (i.e. Λ_*x*_ = Λ_*y*_ → Λ_*xy*_) ranging from ~280–400 nm. In (e), under unpolarized incident light, the variation in analyzer rotation reveals the polarization dependent spectral filtering properties. A range of vivid colors across the visible are observed, which for orthogonal polarization conditions are symmetric about L_*x*_ = L_*y*_, as shown through the color symmetry in the optical images in (e). Hence, the reflectivity map in (d) will be transposed upon orthogonal analyzer conditions, resulting in polarization specific color properties. The optical properties are characterized through the reflectance spectra in (d) - whereby the spectra is recorded with analyzer aligned with L_*x*_. Each plot in (d) represents one of the pixels in the 6 × 6 array (with L_*x*_ and L_*y*_ variations), and each plot consists of 5 sub-plots representing the symmetrical unit-cell spacing variations. Hence, within each 6 × 6 array, anisotropy (aspect ratio) is increased in both x and y, and each 6 × 6 array (out of 5, A–E) representing a different unit-cell spacing (indicated in the Figure).

The results in Fig. 4(d) show that it is possible to achieve both dual and single resonances in order to tailor the reflection spectra across the visible part of the spectrum. As the grating period, Λ_*xy*_, increases (A–E), the resonant modes increase in wavelength, and in combination with increasing anisotropy, eventually split into multiple modes arising from the plasmonic MIM structure - more detail on the nanostructure modes are found in the Supplementary Material. From left-to-right (e), we can observe two short wavelength modes to begin with (i), slowly separate (ii–ii), then transition into a single mode (v–vi), which then eventually begins to split. Due to the spectral position of the shorter wavelength mode, which increases in wavelength as the spacing increases, it can be considered the grating-coupled SPP mode. The longer wavelength mode, which increases in wavelength as the spacing increases, is a hybrid SPP-QGM - more details in Supplementary Material. A wide color gamut is readily available by selecting various geometries i.e. based on the spectra in (d), it is relatively straightforward to generate a wide range of colors in reflection. Hence, in (g) we select three arrays to indicate RGB color properties. For comparison, selected reflectance spectra, from Figs 3(f) and 4(g), are plotted on CIE 1931 chromaticity diagrams in the Supplementary Material.

### Plasmonic MIM Gaussian pixels

The polarization-dependent, high reflectance, spectral control from the nanostructured plasmonic metapixels opens up the possibility of creating more innovative pixel designs than just RGB filter arrays. A conventional high-resolution pixel reflector is normally just single color and with rectangular form-factor, mainly for ease of manufacturing. Yet because microdisplays are used for a plethora applications, this pixel shape may not be optimal for information discretization and display. We propose and demonstrate here the use of graded-pixels, whereby the general in-plane shape and color can be polarization controlled. Here, we utilize in-plane Gaussian profile pixels, shown in Fig. 5. The dimensions of each nanostructure is controlled in 1 or 2D with a Gaussian function. Therefore, the pixel color function is now non-uniform, non-rectangular and hence the sub-pixel spatial profile varies depending on the color.

In Fig. 5 we show the SEM and polarizing optical microscope images (under a range of conditions) of Gaussian pixel profiles - taken from a larger matrix, shown in the Supplementary Material. Different polarizations correspond to different pixel color/spatial functions. That is, a pixel function with polarization and wavelength dependent 2D profiles across the pixel. Moreover, gradient RGB color profiles are obtained across a single pixel, shown in the optical microscope images in (b and d). With specific reference to the intensity plots in Fig. 5(d–i), we isolate the RGB channels of the captured image and show that for one pixel, there are varying 2D profiles for each wavelength (which is polarization controlled). In (e–f) we combine Gaussian and Gaussian-derivative profiles for orthogonal polarizations to show the potential for unconventional color pixel reflector designs - the larger set is shown in the Supplementary Material. This type of pixel, whereby the effective pixel shape and diffraction pattern (far-field) is controlled with an in-plane function encoded with nanostructures on the MIM reflector itself is an extremely attractive toolbox parameter for optical display engineers. Specifically, in designing future holographic display devices, which require a large amount of optical information to be available for modulation, per pixel, in order to form arbitrary optical field profiles^7, 22^. More importantly, because the diffraction pattern (far-field) of a display pixel is related to its aperture function by a Fourier transform, by giving the pixel an in-plane function we can dictate the diffraction pattern of the pixel. Specifically, the far-field of a typical pixel shape (squircle) is sinc-like in nature^43^, resulting in unwanted higher-orders, yet a Gaussian, whose Fourier transform is yet another Gaussian, may offer an attractive solution to suppressing these artifacts.

## Conclusion

In summary a range of nanostructured plasmonic metapixel designs have been demonstrated which offer unique spectral (full RGB) and polarization control for use as novel pixel elements in state-of-the-art high-resolution reflective microdisplays. The designs, composed of ultra-thin Ag, SiO_2_ and Al back-reflector, exhibit a multitude of associated resonant absorption modes including surface plasmon and quasi-guided modes. Through simultaneous excitation of resonant modes, spectrally adjacent to the wavelength of interest, color filtering is achieved and the anisotropic nanostructured elements yield polarization control. We extend the concept to both 1D and 2D nanostructures for dual-resonant behavior leading to highly vivid color pixel profiles. Furthermore, with isolated nanostructures, we can tailor additional 2D (Gaussian) profiles across each pixel which have unique 2D functions for different wavelengths. Because the effects here can be polarization controlled, the integration with liquid crystals (switchable waveplates) makes them promising candidates for a plethora of microdisplay applications and technologies. This design method is compatible with existing manufacturing techniques, for example, aluminium is already the material of choice for existing pixel-reflectors and SiO_2_ is easy to deposit and long-lasting. Initially the work here eliminates the requirement for pigment based filters and input polarizers for reflective display pixels, yet nanoplasmonic MIM pixels (metapixels), which have in-plane spatial profiles are now not limited by the rectangular form-factor of the conventional pixel. This offers the potential to re-imagine the way in which one considers what is meant by a ‘single-pixel’.

## Methods

### Experimental

Pattern designs are produced parametrically in MATLAB and output to a usable patterning format for EBL. Si-SiO_2_(300 nm) samples are cleaned in an ultrasonic bath of acetone for 20 min, 10 min isopropyl-alcohol (IPA), blow-dried with compressed N_2_ and dehydrated at 200 °C for 20 min. 100 nm Al followed by a 100 nm layer of SiO_2_ are deposited using a remote plasma RF sputtering system at a low deposition rate, to form the insulator-back reflector above the Si. This deposition technique ensures close-packing of the deposited material, improving mechanical and chemical stability, along with optical performance. PMMA A4 950K photoresist is spin-coated on top of the sample at 5,500 rpm for 45 s to form a ~150 nm layer, then baked at 180 °C for 2 min to remove the solvent. Electron beam lithography (EBL) (Nanobeam Ltd.) is used for the high-resolution patterning at a range of dose exposures and currents (80 kV acceleration voltage, 1500–2100 *μ*C cm^−2^ Area dose, 1 nAs^−1^ current, main-field aperture 50 *μ*m). A cold-development (T < room temp) step is used with a 1:3 MIBK:IPA solution, for ~10 s, followed by N_2_ blow dry. Deposition of Ag (30 nm) is performed using a thermal evaporator (base pressure 1 × 10^−6^ mbar); rate of ~1 A/s. Resist lift-off is carried out in N-Methyl-2-pyrrolidone (NMP) at an elevated temperature of 60 °C, with 4 hours soaking, followed by fresh NMP sonication for 1 min, acetone rinse, IPA rinse and N_2_ blow dry.

Samples are optically characterized using an Olympus BX-51 polarizing optical microscope, with halogen bulb light-source (IR filters removed for reliable spectra across 400–800 nm), attached to a spectrometer (Ocean optics UV-VIS HR2000+) with a range of objective lenses (10–100x). Reflection results are normalized to a bulk aluminium mirror in the focal plane of the respective objective lens. A Carl-Zeiss scanning electron microscope (SEM) at an acceleration voltage of 1–5 keV is used for imaging the surface of the samples.

### Simulation

Full-wave finite-difference time-domain (FDTD) modeling^40^ is performed. Either periodic or symmetric-anti-symmetric boundary conditions (depending on geometry) are used (x-y boundaries of the unit cell) and perfectly matched layers (z-boundary) along with direction of propagation. For the 1D grating arrays, a 2D simulation environment is used, and for 2D nanostructure arrays, full-3D simulations are used. A uniform cubic mesh (Yee-cell) with dimensions <1 nm and broadband-pulse plane-wave (350–1000 nm) injection sources at a significant distance above the sample are used. For the E-and-H-field intensity plots, an additional finer mesh is included, whereby the smallest cubic mesh size is <0.01 nm (z-direction). Complex dispersive material models are used for silver (CRC model), aluminum (Palik) and SiO_2_ (material data). Reflection values are calculated from power monitors positioned above the range of structures and source injection. The E-and-H-fields are calculated from frequency-power-profile monitors intersecting the structures across 3-planes, processed in MATLAB and plotted.

## Electronic supplementary material

Supplementary Material

RGB metapixels: importance of MIM (unpolarized input)

RGB metapixels: importance of MIM (polarized input)

Symmetrical metapixels with analyzer rotation

Non-square (Gaussian) geometry metapixels 1

Non-square (Gaussian) geometry metapixels 2

Non-square (Gaussian / Laplacian) geometry metapixels 3
