# Supplementary material for: Nanostructured plasmonic metapixels
Source: Sci Rep. 2017 Aug 10;7:7745. doi: 10.1038/s41598-017-08145-0 (PMC5552795; doi:10.1038/s41598-017-08145-0)
Supplement: Supplementary file 1 — Supplementary Material [file 41598_2017_8145_MOESM1_ESM.pdf]

Nanostructured plasmonic metapixels

Calum Williams[1,*], Girish Rughoobur[2], Andrew J. Flewitt[2], and Timothy D. Wilkinson[1]

[1]Centre of Molecular Materials for Photonics and Electronics, Electrical Engineering Division, Department of Engineering, University of Cambridge, 9 JJ Thomson Avenue, Cambridge CB3 0FA, United Kingdom
[2]Electronic Devices and Materials Group, Electrical Engineering Division, Department of Engineering, University of Cambridge, 9 JJ Thomson Avenue, Cambridge CB3 0FA, United Kingdom
[*]cw507@cam.ac.uk

ABSTRACT

In this supplementary material we provide the background to the '*Nanostructured plasmonic metapixels*' manuscript in the form of a larger set of analytical, simulation and experimental results. The work here should be considered in parallel to the main manuscript for completeness.

1 Continuous MIM layer FP-cavity

To light normal incidence upon a continuous metal-insulator-metal (MIM) cavity, the structure acts as a Fabry-Perot filter but with the etalon terminated by lossy mirrors (metals at optical frequencies)[1,2]. Through the modification of insulator and top-layer thickness, highly efficient notch-filtering can be achieved - with reference to the FDTD simulations shown in Figure 1. The top metal layer is ultra-thin (30nm), the dielectric spacer varies which changes the total optical-path-length and contributes to the cavity Q-factor. The top and bottom layers act as lossy mirrors due to the lossy properties of metals at optical frequencies. Changing the dielectric thickness and top-layer thickness changes the Fabry-Perot cavity mode (CM) of the structure. For the structure in the main manuscript, the CM is around 420 - 430nm. At resonance, the E-field is highly concentrated in the central region of the cavity. For a back-mirror thickness greater than the metallic skin depth (~tens of nanometers), narrow-band perfect absorption can be achieved, whereas for thickness similar to the top mirror, transmission filtering is possible.

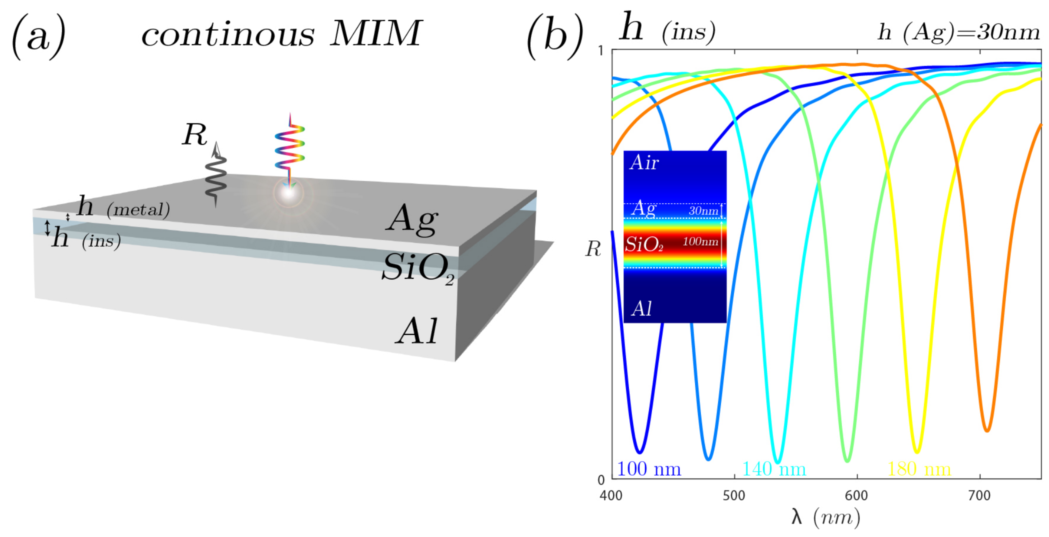

Figure 1. Fabry-Perot MIM notch filter (a) Schematic of continuous MIM structure. (b) Reflection results from varying the the insulator thickness, with E-field magnitude profile inset

2 Nanostructured MIM modes and plasmonics

The transition from continuous to nanostructured ultra-thin top-layer in the MIM geometry allows the tailoring of the absorption properties and opens up the ability to excite a range of different modes. In order to excite SPPs, a phase-matching condition must be met[3,4]. Because SPPs propagate parallel to a metal-dielectric interface (parallel wavevector), the wavevector of incident light must be coupled to a vector-component along this direction in order for SPP excitation. Hence, for light at normal incidence, to provide this extra momentum, we use periodic arrays of nanostructures i.e. the grating vector provides the additional component.

Grating-coupled SPPs

The introduction of 1D or 2D periodicity, allows the coupling of the incident momentum into a tangential component (through diffraction, into a range of orders), which permits the excitation of SPPs. Essentially, the periodic structure provides an in-plane wavevector increase by a factor (1D) of $2\pi j/\Lambda$, where j is an integer multiplier thus allowing the excitation of SPPs at normal incidence. Strong coupling between SPPs and the diffracted modes occurs when eqn. 1 is satisfied,

$$k_{SPP} = k_{\parallel} + k_G \tag{1}$$

$$k_{SPP} = k_0 \left[\frac{\varepsilon_m(\lambda).\varepsilon_d(\lambda)}{\varepsilon_m(\lambda)+\varepsilon_d(\lambda)} \right]^{\frac{1}{2}}, \qquad k_G = \pm m\frac{2\pi}{\Lambda_x} \pm n\frac{2\pi}{\Lambda_y}, \qquad m,n = 1,2\ldots N \tag{2}$$

where $k_{\parallel} = k_0 \sin\theta_{xy,inc}$ is in incident wave-vector (free-space), $k = k_0\varepsilon^{\frac{1}{2}}$ and $k_0 = \omega/c = 2\pi/\lambda$. k_{SPP} is the wave-vector for a SPP between ε_m and ε_d. The real part of the propagation constant determines the SPP wavelength, which is always smaller than the light wavelength in the dielectric $\lambda_{SPP} = 2\pi/\Re(k_{SPP})$. The dispersion relations for SPPs (Ag and Al) are shown in Figure 2. Thus, by sweeping Λ it's possible to intersect the associated SPP dispersion and excite SPPs.

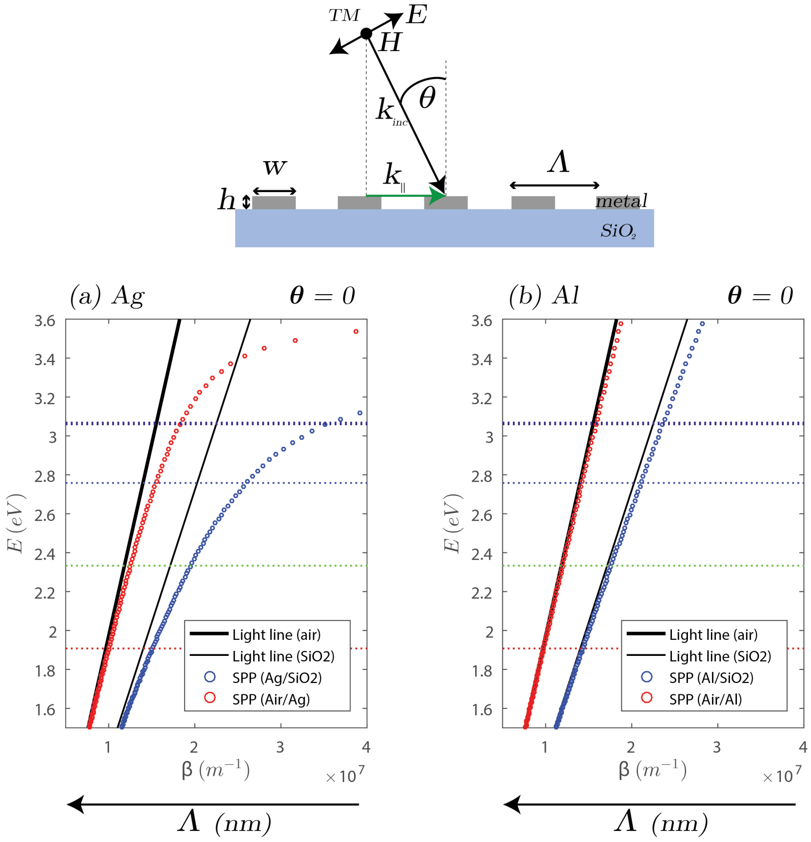

Figure 2. Metal grating upon dielectric. (top) Schematic of grating and labeled parameters. (below) Dispersion relation for SPP based on equation (2) for silver (a) and aluminium (b) at normal incidence

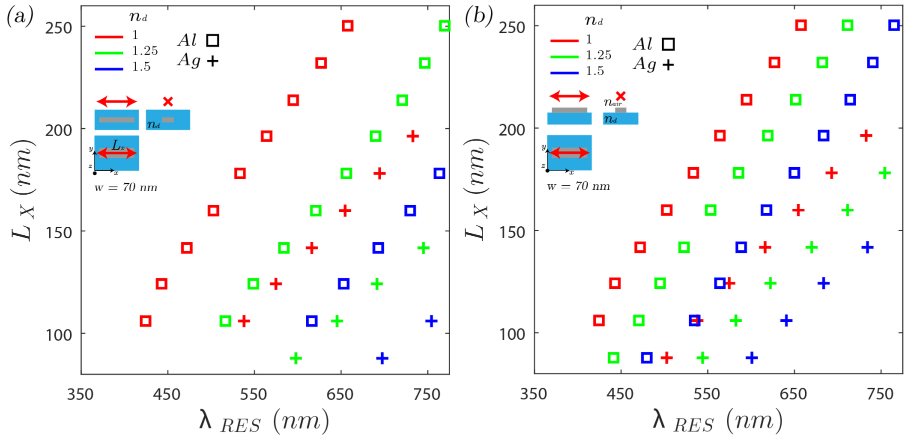

Figure 3. LSPR of arrays of nanostructures: the resonant mode peak wavelength associated with the LSPR taken from scattering-cross-section FDTD simulations in a range of isotropic dielectric background indices (a) and on top of a substrate (b) with the same refractive index iterations.

Localized resonances

The 1D gratings offer singular polarization and wavelength selectivity, however, we can open up a second characteristic by utilizing isolated anisotropic nanostructure arrays. Isolated nanostructures exhibit localized modes: resonant modes confined to individual metal structures[3–5]. When the mean-free-path of the metal's electrons is greater than the actual physical dimensions of a metal, a quantization of the plasmon frequency occurs. This is a collective charge density oscillation of the conduction electrons which has been excited: referred to as a localized surface plasmon resonance (LSPR) (note: the 1D array can also offer localized resonances). Upon illumination, with incident light consisting of a component with the plasmon frequency, a strong extinction (absorption + scattering) of light at that wavelength will occur. Metallic nanoparticles offer large extinction coefficients (energy removed from the system = absorption + scattering) which make them useful in a variety of applications. Mie theory in combination with Rayleigh-Gans theory accurately describe the optical response of isolated, and few system, nanoparticles[3,5]. In the quasi-static limit, the scattering and absorption cross sections, σ_{scat} and σ_{abs}, of an ellipsoid can be defined as

$$\sigma_{scat} = \frac{k^4}{6\pi} \left|\alpha_j\right|^2, \qquad \sigma_{abs} = k \Im \left|\alpha_j\right| \tag{3}$$

where k is the wavenumber, α_j is the complex polarizability of the structure, with $j = a,b,c$, corresponding to three semi-axes of an ellipsoid, where each axis has its own polarizability, with depolarization factor for each axis. Thus, the extinction properties vary depending on the axis (input polarization state). The extinction cross section, σ_{ext}, is a summation of the two. For spheres, $\alpha \propto R^3$, and hence because $\sigma_{abs} \propto R^3$ compared to that of $\sigma_{scat} \propto R^6$, at small particle radii, absorption dominates over scattering. Furthermore, the unit cell and periodicity will have an effect on the optical output and is a summation of the power scattered from each induced dipole[6]

FDTD simulations (Lumerical FDTD solutions) are performed, shown in Figure 3 highlighting the effective linear scaling[7] of Ag nanostructures on different substrates. In free space, the particle scattering resonance is dominated by a LSPR. The presence of an interface breaks the symmetry, and the mode is localized at the interface with the substrate. The efficiency changes and asymmetry is introduced. At short wavelengths, a dipole-like, free space resonance dominates, while at long wavelengths, the resonance can be attributed to the excitation of resonant SPP modes at the particle/substrate interface[3,8].

Guided modes

The proposed pixel geometry (multi-layer system) is such that we have insulator-metal-insulator (IMI) and metal-insulator-metal (MIM) areas, where many different SPP modes can exist. In addition, with a top-layer nanostructure which scatters into a range of k-vectors, and the fact that IMIs and MIMs can act as waveguides (plasmonic / oscillatory modes), we must consider plasmonic waveguiding concepts. It's possible that one or more of the scattered wavevectors can be coupled into a waveguiding mode. The origin of plasmonic waveguides is based on the excitation and waveguiding of SPPs at the interfaces in MIM or IMI geometries[4,9–13]. Due to the symmetry of the MIM structure, generally the associated characteristic dispersion relations split into two equations, describing *symmetric* (long-range SPP (LR-SPP)) and *anti-symmetric* modes (short-range SPP (SR-SPP)).

The characteristic equations of a MIM wave-guiding structure are:

$$\tanh\left(\frac{k_i t}{2}\right) = -\frac{\varepsilon_d k_m}{\varepsilon_m k_d} \qquad i.\ symmetric\ mode$$
$$\coth\left(\frac{k_i t}{2}\right) = -\frac{\varepsilon_d k_m}{\varepsilon_m k_d} \qquad ii.\ anti-symmetric\ mode \tag{4}$$

$$where, \quad k_{m,d} = \left[k_z^2 - \varepsilon_{m,d}\, k_0^2\right]^{\frac{1}{2}} \tag{5}$$

For a MIM structure with small dielectric thickness of $<$100nm (with semi-infinite metal boundaries), only the fundamental mode TM_0 exists[12,14,15]. Note, the TM_0 mode is plasmonic in nature for any dielectric thickness. For a 3-layer metal-insulator-air system, Figure 4 (Al-SiO_2-air), the dispersion relation becomes[16],

$$k_{x2} t_2 = \tan^{-1}\left(K_{21}\frac{ik_{x1}}{k_{x2}}\right) + \tan^{-1}\left(K_{23}\frac{ik_{x3}}{k_{x3}}\right) \tag{6}$$

$$where, \quad K_{ij} = \frac{\varepsilon_i}{\varepsilon_j} \quad (TM)$$
$$k_{xi}^2 = k_i^2 - k_z^2 = \varepsilon_i k_0^2 - k_z^2 \tag{7}$$

The dispersion curve of these guided modes in this asymmetric metal-insulator-air (M-I-A) structure lie in the region between the light line of air and light line of the insulator[14,16,17]. The equations above are solved numerically (MATLAB), and shown in Figure 4 (Al-SiO_2-air). With increasing insulator (material - 2) thickness, the fundamental mode becomes more greatly confined to the insulator, shown through the bending of the dispersion toward the insulator light line (lower energies). The position of this line is key for inference on the data later on in this document.

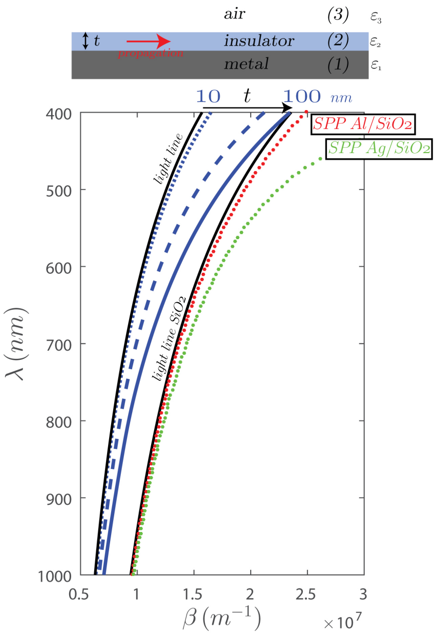

Figure 4. Metal-insulator-air (M-I-A) geometry dispersion. (top) Schematic of the typical geometry and labeling of M-I-A structure. (bottom) Dispersion relations of the guided modes with varying dielectric thickness (with n = 1.5, approximately that of SiO_2, but kept constant in order to rule out the effects based on dispersion within the insulator). Typical SPP dispersions and light lines are also plotted for comparison.

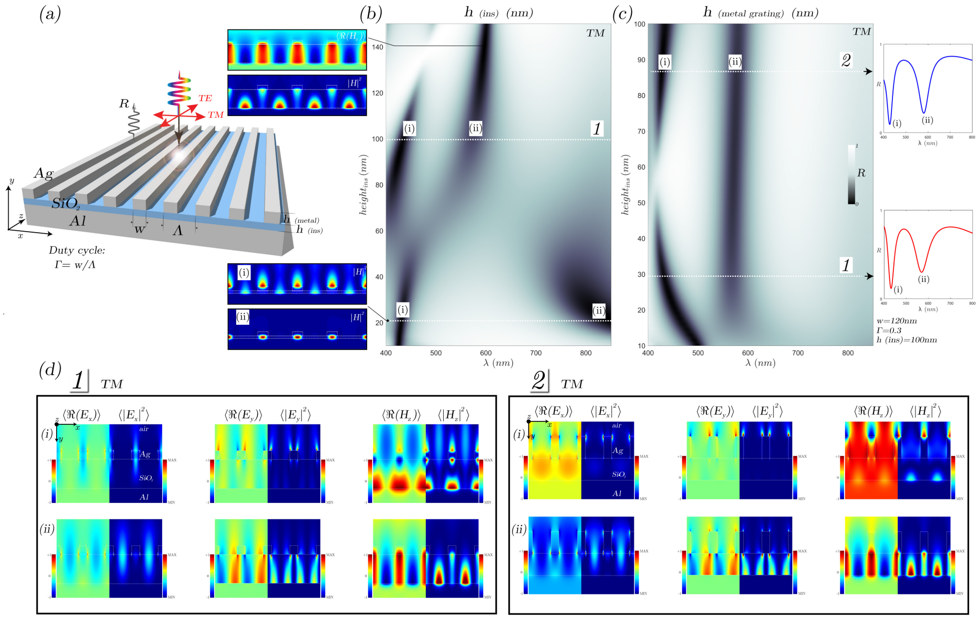

Figure 5. 1D MIM grating reflector (TM) FDTD simulations to determine suitable thickness values. (a) Schematic of MIM grating with indication of parameters and TM-polarization. (b) Increasing insulator thickness with constant top-layer thickness (30nm), with E and H-field intensity profiles represented in insets surrounding the plot. (c) Increasing top layer (Ag) thickness with constant insulator thickness (100nm) with E and H-field plots at specific points in the insets.

2.1 FDTD simulations:

Material thicknesses

To explore the available pixel characteristics, material thickness requirements, required periodicity etc., finite difference time domain (FDTD) simulations[18] are performed. Due to symmetry arguments with the gratings, the simulations are performed in 2D with periodic boundary conditions at the varying unit-cell boundaries. Perfectly matched layers are used above and below the structures. Power monitors are placed above the source injection plane to monitor reflection response. Complex dispersive material modes (Palik and CRC) are used for aluminium and silver. A fine mesh <1 nm is used for the entirety of the structure, but a more refined mesh, <0.01 nm (z-axis) encompassing the interfaces.

Firstly, we explore the effect of insulator, and top-layer metal, thickness. Note, the back-reflector, even though it has finite thickness, is considered bulk in all simulations due to the fact that its thickness is much greater than the associated skin-depth. In Figure 5 the insulator thickness and top-metal (Ag) grating thickness is increased, and reflection plotted for a 1D periodic, Ag-SiO_2-Al(100nm)-SiO_2(300nm)-Si(bulk) MIM-pixel-reflector. As the insulator (SiO_2) increases from 10nm to 150nm we observe multiple absorption mode branches. The duty cycle is kept constant at 0.3 for a grating width of 120nm (hence grating vector of 400nm). The insulator thickness is iterated in (b) and the metal grating thickness is iterated in (c). In (b) as the insulator thickness increases (top metal at 30nm), two modes are present. The shorter wavelength mode is indicative of both SPP (Ag-air) and weak guided mode (Al-SiO_2), due to the H-field intensity profile and reference dispersion to relation in the above section. The longer wavelength mode resembles that of a gap-SPP (G-SPP), due to the highly localised H-field profile bottom reflector and just the grating. As thickness increases, we then see this shorter wavelength mode split and two absorption modes appear at approx. 60nm. The shorter wavelength mode here, 1(i), exhibits both SPP (Ag-air) and (Ag-SiO_2) intensity profiles and the longer wavelength absorption (ii) resembles a hybridized mode, as both SPP (Ag-SiO_2) and quasi guided-mode (QGM) TM_0 behavior is present. Because incident light (TM) has no component in the y-direction, the middle column plots in (1) and (2) indicate that for the longer wavelength modes (ii), there are E_y components in the structure. These field patterns are indicative of TM_0-guided plasmonic modes, in combination with the top layer metal-dielectric SPP. Hence, there is a coupling between SPPs and waveguiding modes, thus leading to the inference of waveguide-plasmon hybridization. For the top-layer metal thickness iteration sweeps in Figure 5 (c), for the larger thickness, the shorter mode is strong SPP excitation (Ag-air) and the longer is QGM behavior once more.

Based on the sections above, for the experimental work and latter parts of this section, we use 30nm top layer Ag, and 100nm SiO_2. This is based on the compromise between an ultra-thin device, optimal performance for dual-resonance (minimum) behavior, limiting just one QGM etc. i.e. we want a system whereby, a few modes are present and can be controlled in a

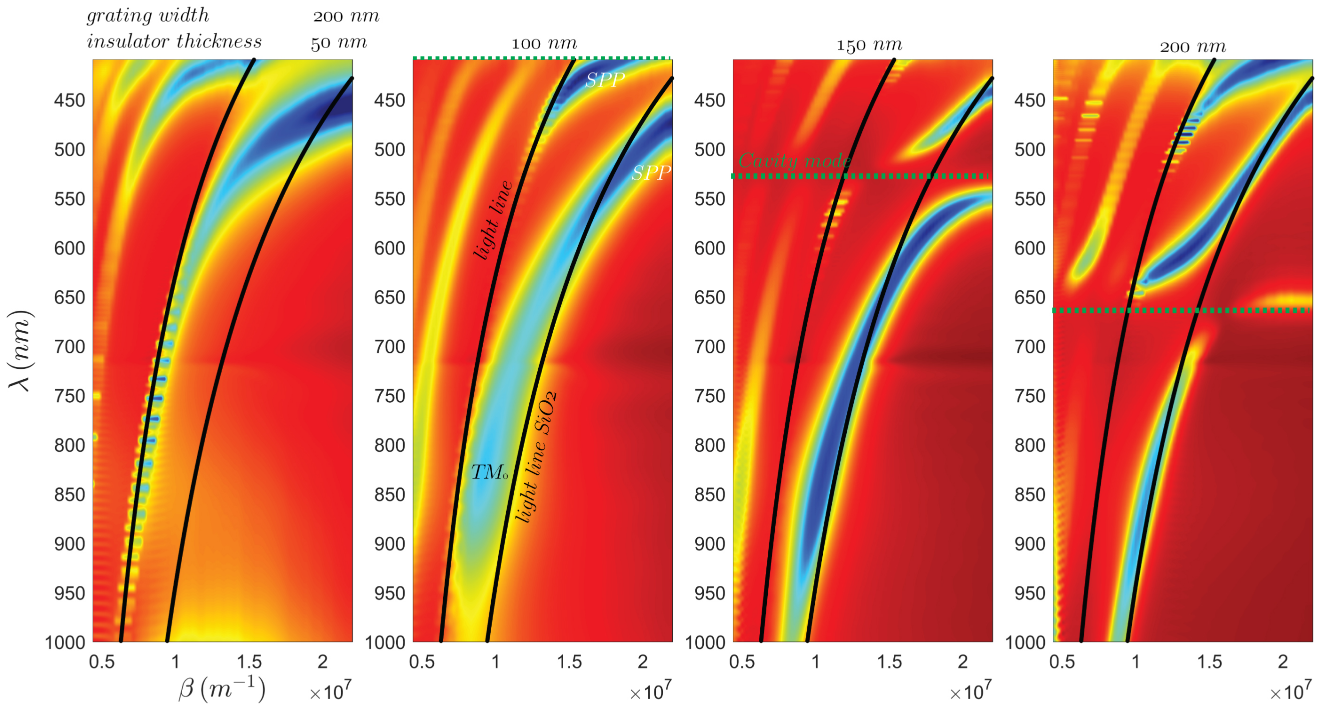

Figure 6. MIM FDTD-simulations: reflection with varying duty cycles (in-plane grating vector sweep) with varying insulator thicknesses. (left-right) increasing SiO2 thickness from 50 - 200nm. The top layer metal thinkness is 30nm. Overlaid are the light-lines in air and in SiO2 and identification of the cavity modes (CMs) associated with the Fabry-Perot behavior (reference to Figure 1).

relatively straightforward fashion. For example, if we were to increase the insulator thickness, additional guided-modes can be excited (hence additional absorption branches), which is both attractive for future devices, but for this work, is unwanted in order to prove the concept.

FDTD dispersion analysis

We now demonstrate the optical properties (in relation to dispersion properties in the above sections) of a nanostructured periodic MIM system through FDTD simulations of varying insulator thicknesses Figure 6, and a range of grating types Figure 7. For Figure 6, whereby the insulator thickness is iterated from 50 - 200nm, with constant grating (Ag) width (200nm) and thickness (30nm), and back-reflector of Al. The plots are represented as a energy (λ) vs. in-plane wave-vector (β), and have overlaid dispersion plots from above sections for ease of reference. The cavity modes linearly scale with increasing insulator thickness (terminated by a lossy top-layer mirror) as shown in Figure 1, and are subsequently plotted on the graphs. We see that between the QGM mode and the cavity mode line, there is anti-crossing behavior. The QGM is tight to the light-line (air) with small insulator thickness and decreases in energy (moves towards the insulator light line) with increasing thickness, as shown in analytical section above - Figure 4. Moreover, the choice of 100nm as the insulator thickness means we have the attractive potential to terminate the longer wavelengths in reflection, with correct choice of grating width and duty cycle. Toward the left of the light-line (air) we see the absorption due to higher order diffractive modes, which are not investigated here.

For Figure 7, we have 3 different grating types (different rows in the figure) with varying grating widths (100-150-200nm) for the columns. Each plot is the reflection response sweep for varying duty cycles and typical SPP and light-line dispersions are plotted over the top for reference. For the top-row (essentially the system we utilize in the main manuscript), typically going from right-to-left (x-axis) the duty cycle decreases from continuous layer to periodic. We start with the fundamental cavity mode resonance. From the simulations in Figure 1, this cavity mode (100nm insulator) is around 420nm. Note, the QGM or SPP dispersion relations do not naturally terminate here toward the larger duty cycles, it's because (as seen with Figure 6) there is anti-crossing behavior here. From diffraction grating theory, generally as the periodicity (in-plane vector) increases, the number of diffracted modes increases. Therefore, because of the increase in in-plane momentum arising from the grating, we can now couple a scattered wave into the plasmonic guided-mode. In Figure 7 (top-row), in relation to the dispersion and E-and-H-field profiles, (a) and (b), mode (i) resembles that of SPP excitation, mode (ii) is indicative of both SPP and QGM

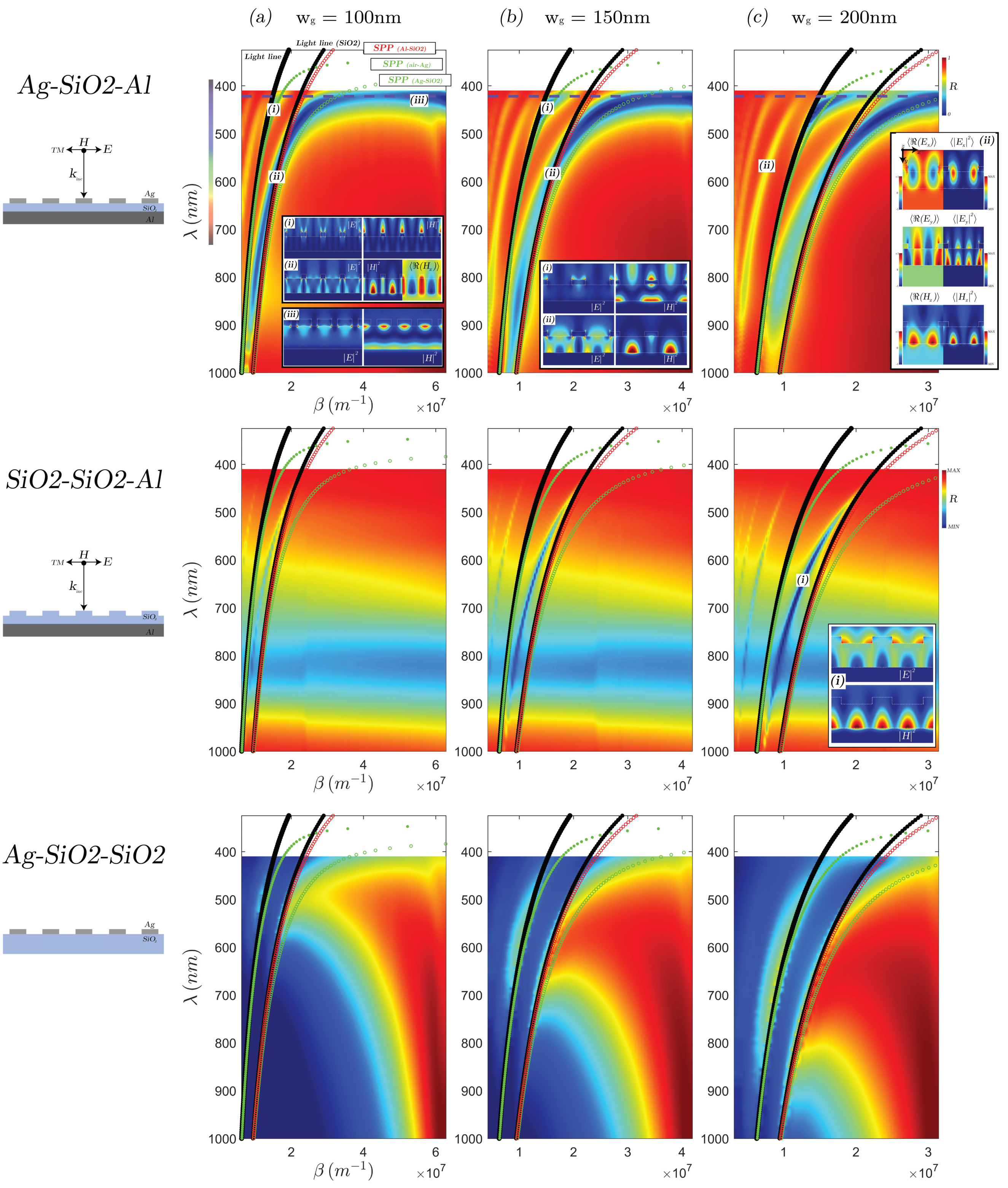

Figure 7. FDTD simulations of the reflection response sweeps for 3-types of grating, with varying grating widths. (top-row) Ag(30nm)-SiO_2(100nm)-Al(bulk) grating, with insets of the E and H-field intensity profiles at the points labeled on the plots. (middle-row) SiO_2(30nm)-SiO_2(100nm)-Al(bulk) grating (hence all dielectric periodicity). (bottom row) Ag(30nm)-SiO_2(bulk) grating, i.e. no back reflector in the system

behaviour, and mode (iii) resembles SPP and cavity-mode behavior. This can be supported by the mode profile in (iii) which is where the second branch tends to with increasing duty. However, mode (i) also resembles LSP behavior with Ag-air. Mode (ii) resembles that of SPP-wave-guiding modes. As the grating vector increases (duty cycle decrease), we start off with the SP mode (Ag-SiO_2). To prove we have guided-modes, the (middle-row) shows an all-dielectric grating (which should yield diffracted modes into the insulator) with Al back-reflector (bulk) reflector. We observe an absorption mode (QGM) between the two light lines which is indicative of the TM0 guided mode. In addition, the inset in the (c) shows the near-field intensity profiles, which resemble the guided mode of the M-I-A structure. Note, this is impossible to achieve without the periodic top-layer and metallic back-reflector, as demonstrated with the (bottom-row) of Figure 7.

Angle dependency simulations

In Figure 8, for the angle-dependent plots, we notice that there are many folded absorption branches from both SPP and guided-modes. This behavior is normally attributed to the structure acting like plasmonic crystal (analogous to photonic crystals). Moreover, these plots are also indicative of guided mode behaviour whereby each guided-mode branch represents varying diffraction orders (m = 1,2,3 etc.). The insulator thickness is 100nm, and with the Ag top-layer, the cavity mode is toward the blue part of the spectrum at around 425nm. Starting with the (top row; grating width 100nm), the large duty cycle shows one absorption mode (cavity mode). With decreasing duty cycle, multiple modes become apparent. These are indicative of both SPP mode behaviour and QGMs of varying diffraction orders[14, 14, 17, 19]. With larger grating width, more branches become apparent. Essentially the plots here add to the claim of guided modes within the structure for the longer wavelength modes, due to the absorption branch behavior at increasing angles of incidence.

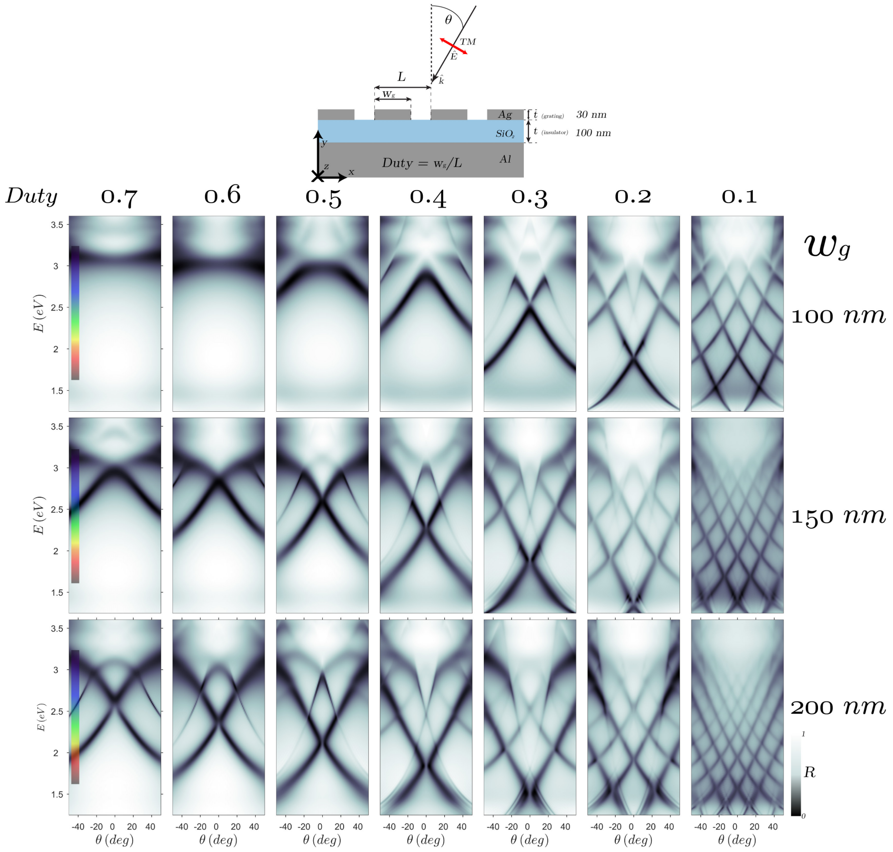

Figure 8. MIM grating reflector angle dependency FDTD simulations: Ag(30nm)-SiO_2(100nm)-Ag(bulk). (top) Schematic of MIM grating showing the structure in relation to incident angle of the TM-input. (bottom) How the MIM-reflector resembles plasmonic crystal absorption properties over a range of incident angles: each row is a different grating width, each column is a different duty cycle (proportional to grating vector), with each reflection map plot as function of incident angle.

2D periodic arrays of MIM-nanostructures

To investigate potential mode profiles within the arrays of nanostructures, we explore both the E and H-field profiles at certain excitation conditions in Figures 9 and 10, and reflectivity response maps with varying nanostructure geometries and spacings, Figures 11 and 12. Now, unlike the the 1D MIM grating, the 2D structure can support both fundamental TE and TM modes, but also lateral Fabry-Perot modes[20,21] where an SPP propagates beneath the nanostructures and can interact with adjacent structures. These are also in addition to the modes described in the previous sections. Moreover, more often than not, the modal profiles resemble elements from different mode types, due to overlapping spectral behavior if one were to plot the dispersion properties. Figures 9 and 10 show the field profiles of the isolated nanostructures with a MIM geometry at the absorption modes. Figure 9 is where $L_x = L_y$. Figure 10 is of an anisotropic rod exhibiting polarization dependent absorption properties, hence polarization dependent spectral filtering properties. For the former, there are two absorption modes. The shorter wavelength mode field maps resemble that of a hybridization between lateral FP-modes underneath the structure and vertical cavity-mode (Fabry-Perot). The longer wavelength mode is indicative of the fundamental QGM and another lateral FP-mode. For the latter (anisotropic geometry), there is one absorption mode for polarization parallel to the long-axis. This mode resembles that of a lateral FP-mode (underneath the rod), LSP and QGM. For orthogonal polarization, two modes are present: the shorter wavelength mode of the two is localized in nature (LSP), and longer wavelength resembles a coupling between SP (metal-SiO_2) and QGM.

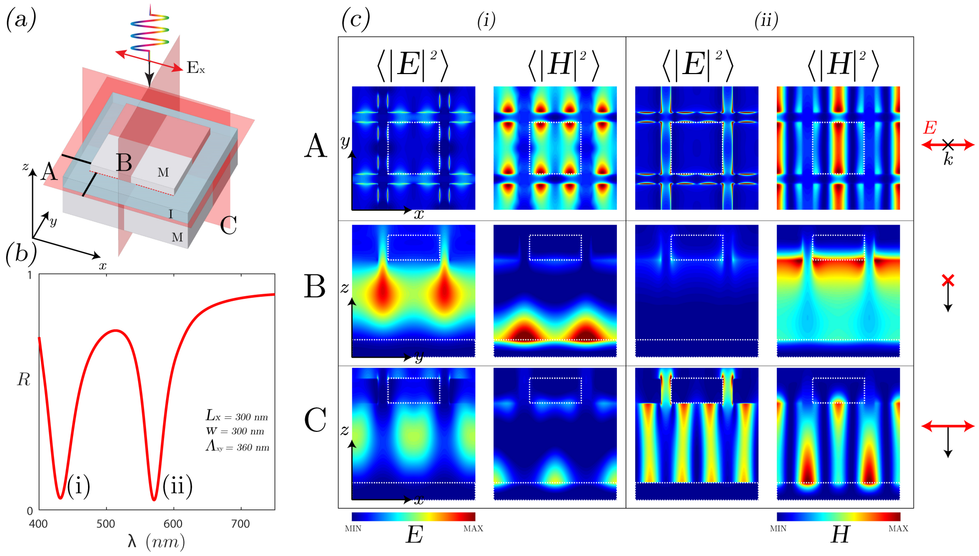

Figure 9. FDTD simulations of square silver nanostructure, MIM geometry: (a) Schematic of structure with indications of where the field cross-sections are taken. (b) Reflection profile of the structure and (c) the E and H-field maps taken at the absorption modes (i and ii) indicated in (b).

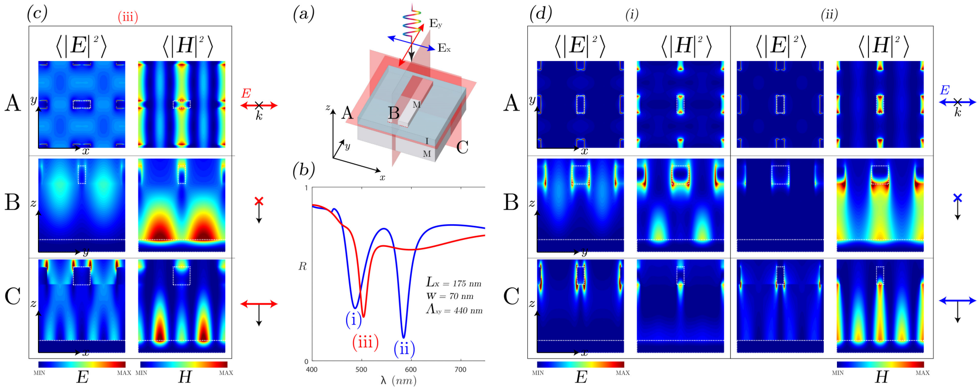

Figure 10. FDTD simulations of anisotropic silver nanostructure, MIM geometry: (a) Schematic of structure with indications of where the field cross-sections are taken. (b) Reflection profile of the structure under the two orthogonal polarization conditions (c and d) the E and H-field maps taken at the absorption modes (i, ii, iii) for the two orthogonal polarizations.

Figures 11 and 12 show the reflectivity maps of a range of 2D nanostructured MIM structures, where the spacing is iterated Figures 11 with fixed nanostructure geometry and vice-versa in Figures 12. For the former, we fix the symmetric unit-cell to 360 nm, and vary the nanostructure geometry. The following plots show that through the suitable choice of geometrical parameters, one can excite both singular and multi-mode absorptions to tailor for user desired spectral properties. In Figure 11 (c) starting from the left (i), we observe the LSP mode starting from approx. 550nm and increasing in resonant wavelength as L_y increases. This mode hybridizes with a lateral FP-mode, which appears toward larger L_y (vi), as L_x increases. There is also a shorter wavelength mode which increases in energy (shorter wavelength) as the x-dimension of the structure increases resembling that of a QGM. In the center part of (c), the energy is distributed between three modes and splitting occurs. In Figure 12 we now have a sweep of the unit cell spacing, with varying anisotropic nanostructure geometries (in the plots typical dispersion curve are presented for ease of reference). In these sub-plots, we observe the interplay between QGMs, both TE and TM, lateral cavity modes, grating coupled SPs and LSPs. Moreover, it is now clear that we have a coupled system with several eigenstates and hence the SPP and cavity modes modes should possess several properties from each other to form a new kind of plasmonic quasi-state[22,23,23–25].

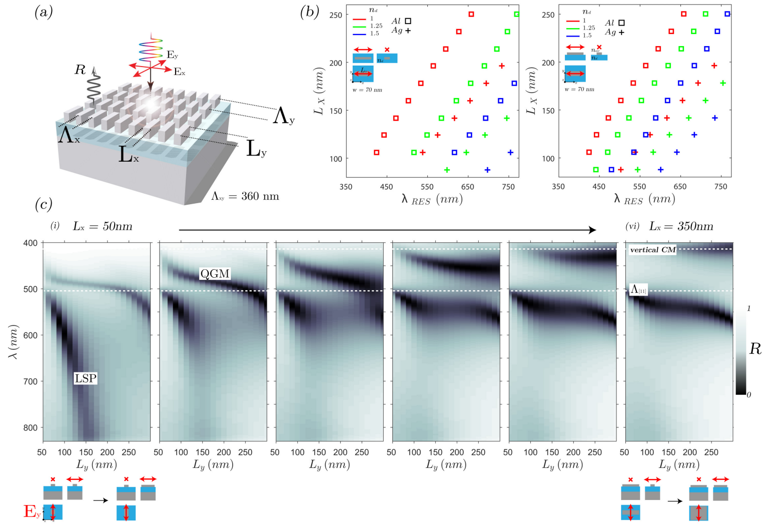

Figure 11. FDTD simulations of 2D arrays of nanostructures in MIM (Ag-SiO_2-Al) geometry: (a) Schematic of typical nanostructured MIM geometry with labeled parameters. (b) LSP resonant peaks plotted with different external conditions for varying sized anisotropic nanostructures. (c) Reflectivity plots of varying geometry MIM structures, with fixed unit cell at 360nm, (i - vi) is the variation in Lx parameter, which is orthogonal to the input polarization

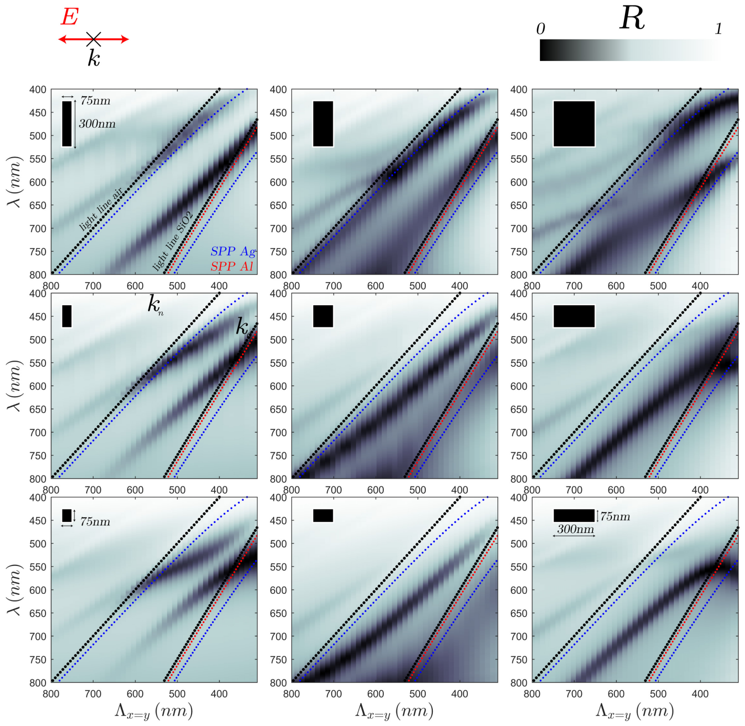

Figure 12. Reflectivity plots from FDTD simulations of varying geometry MIM (Ag-SiO_2-Al) structures (9 in total) with fixed input polarization (indicated in relation to structure on top left) and varying unit cell size. Overlaid for ease of reference are the light-lines in air and SiO2, with the SPP dispersion curves for Ag (air and SiO_2) and Al (SiO_2)

3 Experimental section: plasmonic metapixels

1D gratings: importance of MIM-geometry

Here we demonstrate the critical nature of the MIM-geometry for plasmonic metapixels in comparison to the optical properties of the same nanostructures on conventional SiO_2(300nm)-Si. Figure 13 shows the difference in reflection between the 1D gratings with MIM geometry and without MIM geometry of a variety of 1D gratings under near-crossed polarizers, with incident polarization at 45-degrees to the grating vector of the structures. The distinct colors are from the MIM mode behavior covered in the above sections and concepts in the main manuscript. The image in (a) is on the Ag(30nm)-SiO_2(100nm)-Al(100nm)-SiO_2(300nm)-Si(bulk geometry and in (b) is of Ag gratings on SiO_2(300nm)-Si(bulk). Figure 14 shows the effect of the MIM geometry on the RGB pixels highlighted in the main manuscript. The optical images are under a microscope with polarized incident light and varying analyzer angle. We patterned structures over the edge of the insulator-back reflector in order to observe the effect. The reflected colors on the bare substrate vary from the MIM geometry considerably and are highly reduced in brightness.

For visual demonstration of these effects, please see the included supplementary videos titled:

- **Video** - **Importance of MIM geometry 1** (Legend: RGB pixel designs patterned over a step-edge of the MIM geometry. Input polarization, rotating analyzer reveals the differing colour response between MIM geometry and pure Si-SiO2).
- **Video** - **Importance of MIM geometry 2** (Legend: RGB pixel designs patterned over a step-edge of the MIM geometry. No input polarization, rotating analyzer reveals the differing colour response between MIM geometry and pure Si-SiO2).
- **Video** - **1D reflectance (art deco)** (Legend: Art deco-like pixel designs. Unpolarized input and continuously varying output polarization (analyzer)).

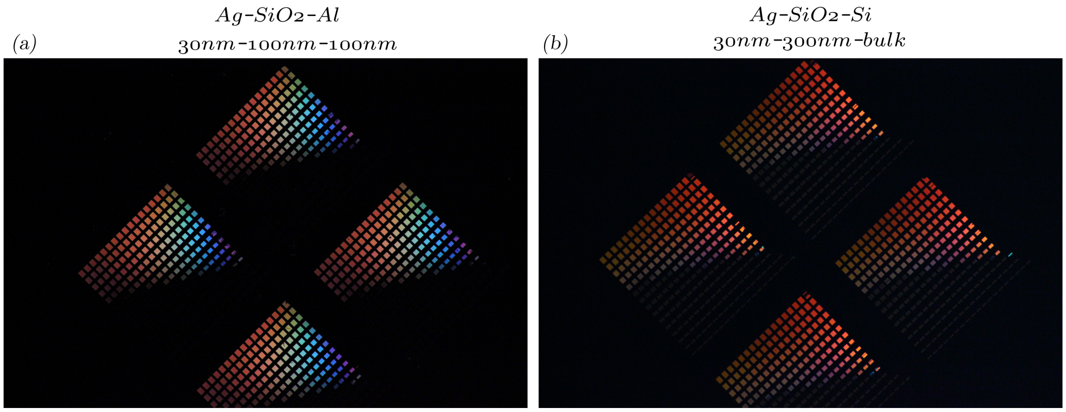

Figure 13. Experimental 1D Ag gratings under near-crossed polarizers: (a) MIM geometry and (b) SiO_2-Si geometry.

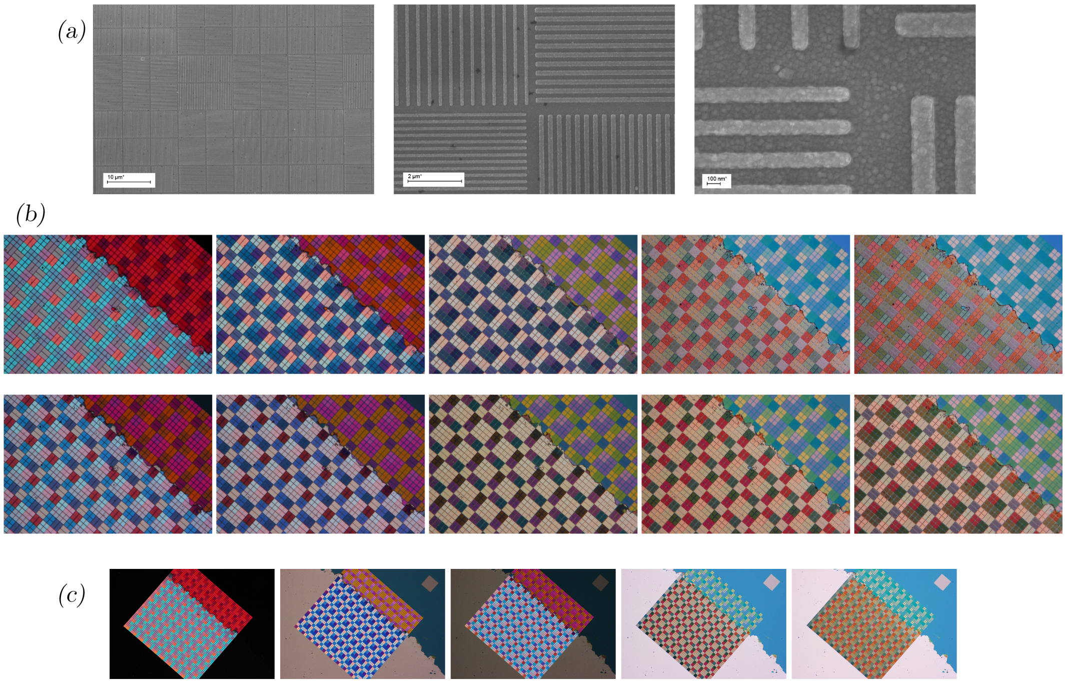

Figure 14. RGB pixel experimental supplementary showing the necessity of the back-reflector and insulator spacer. (a) SEM images of some RGB pixel attempts at varying magnfications, (b) and (c) show the large arrays of the pixels under varying analyzer conditions (optical microscope) with input polarized.

2D arrays

Figure 15 shows the difference between MIM and without MIM layers on the reflection profiles of the 2D nanostructure arrays presented in the main manuscript. The crossed state is with incident polarization at 45° to the image, hence the darkened middle squares whereby Lx = Ly and therefore no anisotropy, hence little to no polarization rotation (darkened). We see how, in comparison to (b) which is just the SiO_2-Si, the difference in output color and importance of insulator-back reflector. Figure 16 shows a selected set of 2D arrays from Figure 3 in the main text, which include the SEM images, reflection spectra and optical microscope images of the color response under orthogonal polarization conditions.

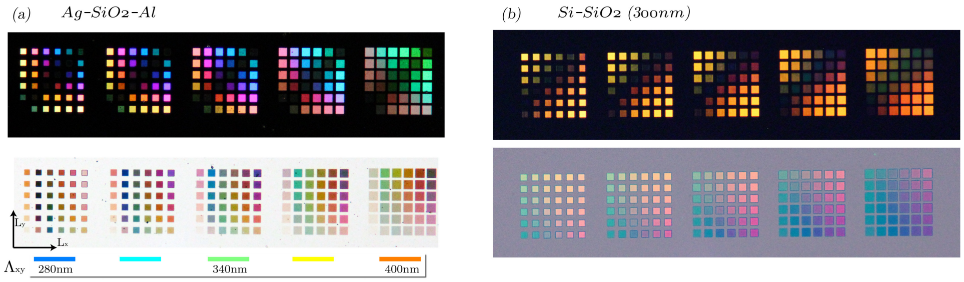

Figure 15. Optical microscope images of fabricated 2D nanostructure arrays with varying periodicity and geometries (in relation to the main manuscript 2D section) for MIM (a) structure and SiO_2-Si (structure). In both (a) and (b) we show the reflection under crossed-polarizers (45 degrees to image) shown in the (top-row) and under unpolarized illumination with output analyzer (bottom-row)

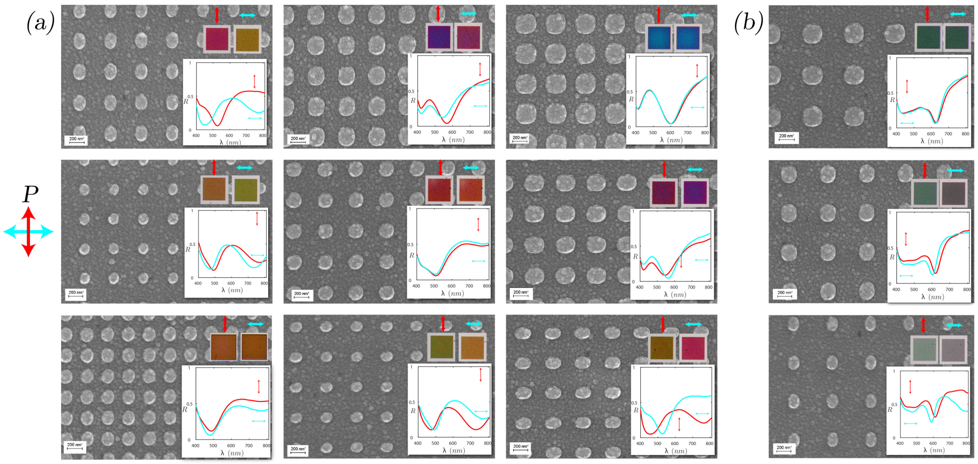

Figure 16. Experimental 2D Ag nanostructure arrays showing the SEM images, optical microscope reflection spectra and images of a range of different arrays, under two orthogonal analyzer polarizations, with unpolarized incident light.

CIE 1931 diagrams

In Figure 3 and Figure 4 (in the main manuscript) shows the reflectance spectra of 1D MIM arrays and 2D nanostructure arrays respectively. Figure 17 shows this data plotted on a CIE 1931 xy chromaticity diagram (alongside sRGB). Figure 17(a) shows the data plotted from Figure 3(d–f) in the main manuscript. For each pixel, orthogonal polarizations reveal either the color (indicated with a cross) or back to Al reflection (shown with a white circle). Figure 17(b) shows the data plotted from Figure 4(g) in the main manuscript – selected from the RGB pixels only (one polarization state).

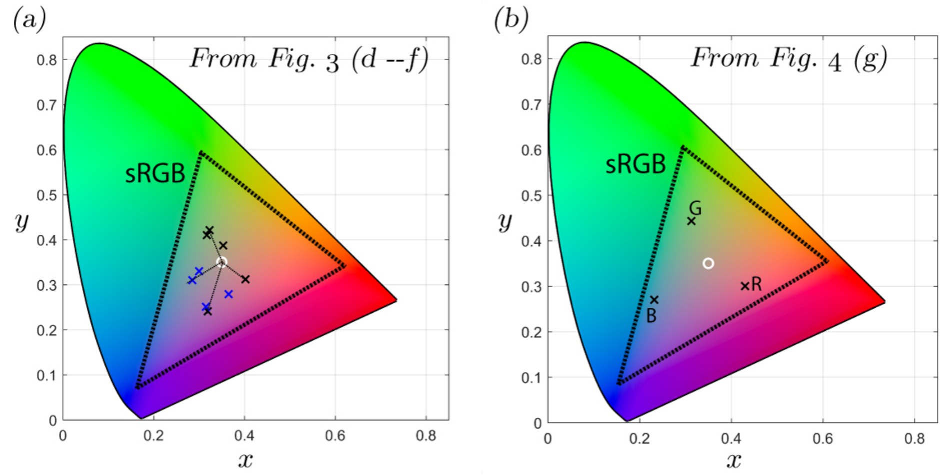

Figure 17. CIE 1931 xy chromaticity diagrams (sRGB profile indicated), for 1D MIM arrays and 2D LSP arrays. (a) Chromaticity diagram for the data in Figure 3 (d–f) in the main manuscript, for the RGB colors under two orthogonal polarizations. The black and blue crosses indicate orthogonal polarizations and the white circle is reflected state. i.e. there are nine crosses in total – for each pixel – whereby rotating the polarization reverts to the reflected state. (b) Chromaticity diagram for the data in Figure 4 (g) in the main manuscript, for the RGB colors from the LSP arrays under one polarization state.

Gaussian profiles

In Figure 18 we show the compete image of Gaussian metapixels from the main manuscript (Figure 4 - main); SEM and polarizing optical microscope images (under a range of conditions) of Gaussian pixel profiles. The top-row shows 2D Gaussian profiles encoded with the MIM plasmonic nanostructures, and bottom 1D. Different polarizations correspond to different pixel color / spatial functions. That is, a pixel function with a polarization and wavelength dependent 2D profile across the pixel. Figure 19 shows the expanded full set of the MIM Gaussian pixels (shown in the final section of the main manuscript and in Figure 18). (a) Shows the 2x 1D Gaussian profiles (for the two axes) under varying polarization conditions and how the color response takes the shape of the Gaussian function. (b) Shows the singular 2D Gaussian profile under varying polarization conditions (a movie of how this appears under varying polarization conditions is included).

Figure 20 shows the fabricated higher-order 2D Gaussian profiles without MIM geometry (i.e. on SiO_2(300nm)-Si(bulk) under SEM and under the optical microscope in crossed polarization and orthogonal output polarizers (with incident polarization). In this Figure, it's clear that the color response is deep rooted in the MIM behavior. Figure 21 shows the full set of the MIM Gaussian-Gaussian derivative pixels for the two orthogonal polarizations (no input polarization) and SEM images.

For visual demonstration of these effects, please see the included supplementary videos titled:

- **Video - Gaussian Metapixels 1** (Legend: Gaussian metapixels under the optical microscope. Unpolarized input, continuously varying output polarization (analyzer)).
- **Video - Gaussian Metapixels 2** (Legend: Gaussian metapixels under the optical microscope. Unpolarized input, continuously varying output polarization (analyzer), (highly magnified)).
- **Video - Gaussian Metapixels 3** (Legend: Two orthogonal Gaussian profile metapixels under the optical microscope. Unpolarized input, continuously varying output polarization (analyzer). Different polarizations reveal different in-plane pixels).

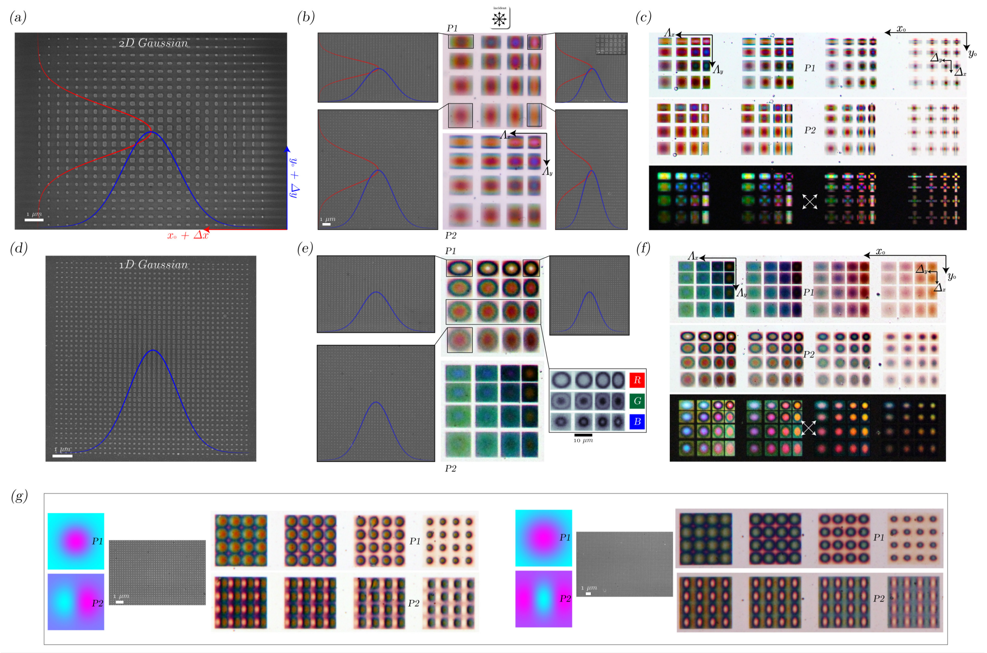

Figure 18. Gaussian MIM plasmonic pixels: (a,d) SEM images of 2D and 1D Gaussian profiled nanostructured pixels, with overlaid Gaussian functions in either x or y. (b, e) Optical microscope images taken under unpolarized incident illumination and orthogonal analyzer conditions, showing gradient color functions associated with each pixel. The insets are of the respective SEM images of the a selection of the pixels. In addition, the inset in (e), is of the RGB channels of one of the Gaussian rows exhibiting how each pixel encodes varying responses depending on the wavelength. (c, f) Show a larger array of the Gaussian pixels under varying analyzer conditions with labeled parameters.

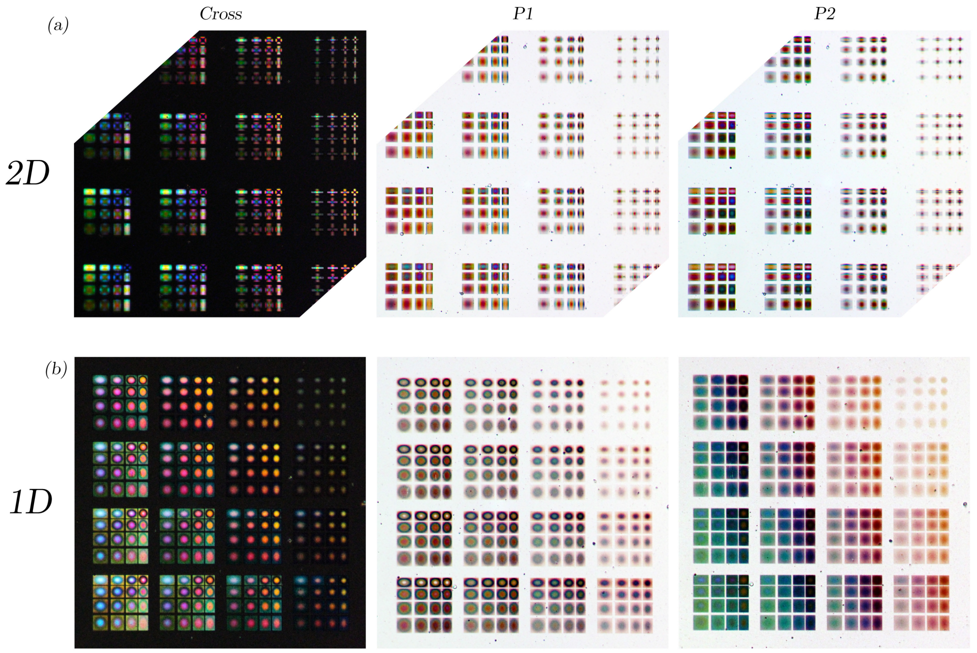

Figure 19. Full set of Gaussian profile pixels: (a) 2x 1D Gaussian profiles (x- y axes) encoded with the 2D arrays of Ag nanostructures (MIM geometry). (b) A 1D Gaussian profile. Both (a and b) show the reflection under the optical microscope for varying polarization conditions

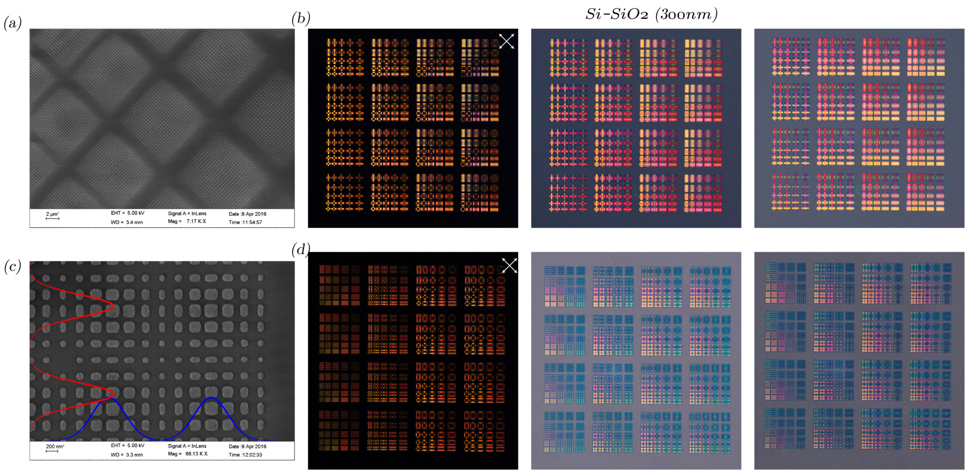

Figure 20. Experimental 2D Ag nanostructure arrays encoded with varying 2D Gaussian profiles on SiO_2-Si (structure). (top-row) is of a more basic Gaussian profile under SEM and under the optical microscope in crossed polarization and orthogonal output polarizers without input polarization. The (bottom-row) is of multiple 2D Gaussian profiles and the as above, the SEM and optical microscope images

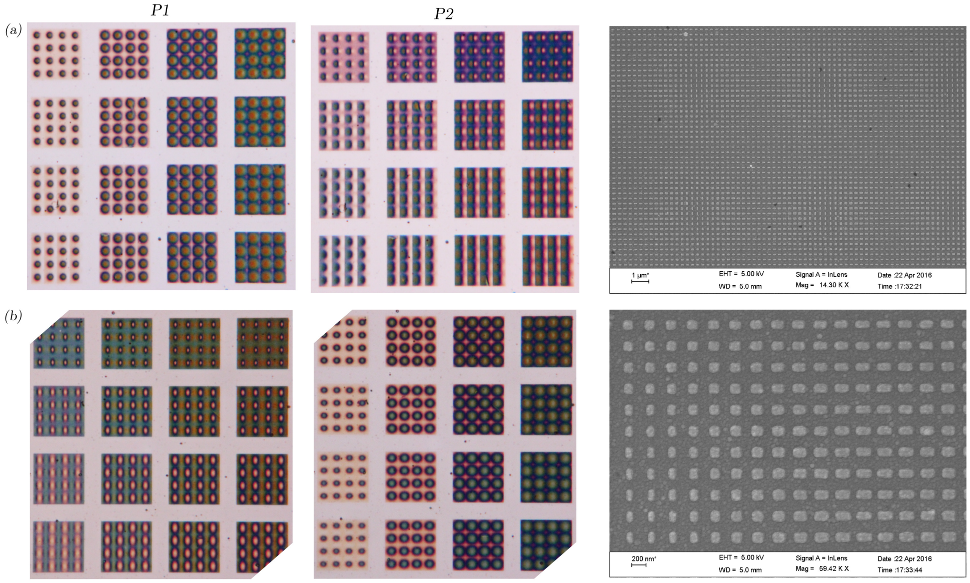

Figure 21. Full set (compared to the sub-set in the main manuscript) of Gaussian-Gaussian derivative MIM profile pixels under varying polarization conditions with SEM images illustrating the in-plane nature of the nanostructured MIM geometry.

References

1. Kajtár, G., Kafesaki, M., Economou, E. N. & Soukoulis, C. M. Theoretical model of homogeneous metal–insulator–metal perfect multi-band absorbers for the visible spectrum. *Journal of Physics D: Applied Physics* **49**, 055104 (2016). URL http://stacks.iop.org/0022-3727/49/i=5/a=055104?key=crossref.c3a75cc1b7854026ac3c68f8103feef9.
2. Li, Z., Butun, S. & Aydin, K. Large-area, Lithography-free super absorbers and color filters at visible frequencies using ultrathin metallic films. *ACS Photonics* **2**, 183–188 (2015). 1410.7792.
3. Maier, S. *Plasmonics: Fundamentals and Applications* (Springer, Boston, MA, 2007).
4. Hayashi, S. & Okamoto, T. Plasmonics: visit the past to know the future. *Journal of Physics D: Applied Physics* **45**, 433001 (2012). URL http://stacks.iop.org/0022-3727/45/i=43/a=433001?key=crossref.d54a1f1e3eb9733ebbdba669bb8ae5e2.
5. Giannini, V., Fernández-Domínguez, A. I., Heck, S. C. & Maier, S. a. Plasmonic nanoantennas: Fundamentals and their use in controlling the radiative properties of nanoemitters. *Chemical Reviews* **111**, 3888–3912 (2011).
6. Olson, J. *et al.* High Chromaticity Aluminum Plasmonic Pixels for Active Liquid Crystal Displays. *ACS Nano* acsnano.5b06415 (2015). URL http://pubs.acs.org/doi/10.1021/acsnano.5b06415.
7. Novotny, L. & van Hulst, N. Antennas for light. *Nature Photonics* **5**, 83–90 (2011). URL http://www.nature.com/doifinder/10.1038/nphoton.2010.237.
8. Beck, F. J., Verhagen, E., Mokkapati, S., Polman, a. & Catchpole, K. R. Resonant SPP modes supported by discrete metal nanoparticles on high-index substrates. *Optics express* **19 Suppl 2**, A146–A156 (2011).
9. Han, Z. & Bozhevolnyi, S. I. Radiation guiding with surface plasmon polaritons. *Reports on progress in physics. Physical Society (Great Britain)* **76**, 016402 (2013). URL http://iopscience.iop.org/article/10.1088/0034-4885/76/1/016402.
10. Davoyan, A., Shadrivov, I. V., Bozhevolnyi, S. I. & Kivshar, Y. S. Backward and forward modes guided by metal-dielectric-metal plasmonic waveguides. *Journal of Nanophotonics* **4**, 43509–43510 (2010). URL http://dx.doi.org/10.1117/1.3437397.
11. Baltar, H. T. M. C. M., Drozdowicz-Tomsia, K. & Goldys, E. M. Propagating surface plasmons and dispersion relations for nanoscale multiplayer metallic-dielectric films. *Plasmonics- Principles and Applications* 136–155 (2012). URL http://www.intechopen.com/books/plasmonics-principles-and-applications.
12. Dionne, J. A., Sweatlock, L. A., Atwater, H. A. & Polman, A. Plasmon slot waveguides: Towards chip-scale propagation with subwavelength-scale localization. *Physical Review B* **73**, 035407 (2006). URL http://link.aps.org/doi/10.1103/PhysRevB.73.035407.
13. Kurokawa, Y. & Miyazaki, H. T. Metal-insulator-metal plasmon nanocavities: Analysis of optical properties. *Physical Review B* **75**, 035411 (2007). URL http://link.aps.org/doi/10.1103/PhysRevB.75.035411.
14. Zhou, W., Suh, J. Y., Hua, Y. & Odom, T. W. Hybridization of localized and guided modes in 2D metal-insulator-metal nanocavity arrays. *Journal of Physical Chemistry C* **117**, 2541–2546 (2013).
15. Lin, C. I. & Gaylord, T. K. Multimode metal-insulator-metal waveguides: Analysis and experimental characterization. *Physical Review B - Condensed Matter and Materials Physics* **85**, 1–9 (2012).
16. Rashleigh, S. C. Four-layer metal-clad thin film optical waveguides. *Optical and Quantum Electronics* **8**, 49–60 (1976).
17. Shi, X.-B., Qian, M., Zhou, D.-Y., Wang, Z.-K. & Liao, L.-S. Origin of light manipulation in nano-honeycomb structured organic light-emitting diodes. *J. Mater. Chem. C* **3**, 1666–1671 (2015). URL http://pubs.rsc.org/en/content/articlehtml/2015/tc/c4tc02596b.
18. Lumerical Solutions, Inc. http://www.lumerical.com/tcad-products/fdtd/. URL http://www.lumerical.com/tcad-products/fdtd/.
19. Wang, S. S. & Magnusson, R. Theory and applications of guided-mode resonance filters. *Applied optics* **32**, 2606–2613 (1993).
20. Kim, W. *et al.* Localized and nonlocalized plasmon resonance enhanced light absorption in metal-insulator-metal nanostructures. *Journal of the Optical Society of America B* **32**, 1686 (2015). URL https://www.osapublishing.org/abstract.cfm?URI=josab-32-8-1686.

21. Marquier, F., Greffet, J., Collin, S., Pardo, F. & Pelouard, J. Resonant transmission through a metallic film due to coupled modes. *Optics express* **13**, 70–76 (2005).

22. Chen, S., Li, G., Lei, D. & Cheah, K. W. Efficient energy exchange between plasmon and cavity modes via Rabi-analogue splitting in a hybrid plasmonic nanocavity. *Nanoscale* **5**, 9129–33 (2013). URL http://www.ncbi.nlm.nih.gov/pubmed/23913114.

23. Zhou, W. *et al.* Extraordinary optical absorption based on guided-mode resonance. *Optics letters* **38**, 5393–6 (2013). URL http://www.ncbi.nlm.nih.gov/pubmed/24322266.

24. Tan, C., Simonen, J. & Niemi, T. Hybrid waveguide-surface plasmon polariton modes in a guided-mode resonance grating. *Optics Communications* **285**, 4381–4386 (2012). URL http://dx.doi.org/10.1016/j.optcom.2012.07.027.

25. Manceau, J. M. *et al.* Mid-infrared intersubband polaritons in dispersive metal-insulator-metal resonators. *Applied Physics Letters* **105**, 1–5 (2014). 1606.05090.
